# Symmetrical and Asymmetrical Thiophene-Coumarin-Based Organic Semiconductors

**DOI:** 10.1021/acsomega.3c05602

**Published:** 2023-12-08

**Authors:** Sinem Altınışık, Mücahit Özdemir, Arzu Kortun, Yunus Zorlu, Bahattin Yalçın, Baybars Köksoy, Sermet Koyuncu

**Affiliations:** † Department of Chemical Engineering, Çanakkale Onsekiz Mart University, Çanakkale 17020, Turkey; ‡ Department of Chemistry, Marmara University, İstanbul 34722, Turkey; § Department of Chemistry, Gebze Technical University, Kocaeli 41400, Turkey; ∥ Department of Chemistry, Bursa Technical University, Bursa 16310, Turkey

## Abstract

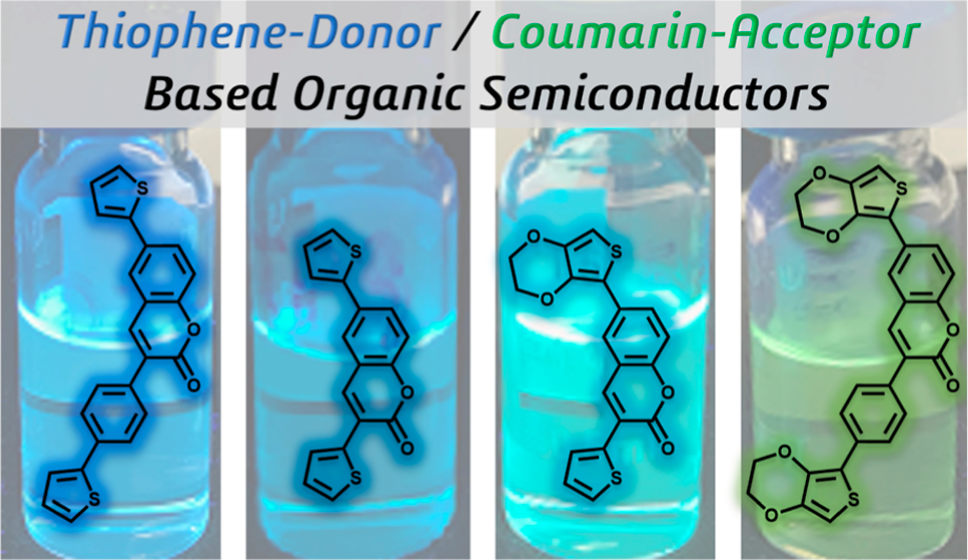

Organic semiconductors are a valuable material class for optoelectronic applications due to their electronic and optical properties. Four new symmetric and asymmetric thiophene-coumarin derivatives were designed and synthesized via Pd-catalyzed Suzuki and Stille Cross-Coupling reactions. Single crystals of all synthesized thiophene-coumarin derivatives were obtained, and π···π interactions were observed among them. The π···π interactions were supported by UV–vis, transmission electron microscopy, and atomic force microscopy analyses. The photophysical and electrochemical properties of the coumarins were investigated and supported by density functional theory studies. Fluorescence quantum yields were recorded between 36 and 66%. Moreover, mega Stokes shifts (175 nm or 8920 cm^–1^) were observed in these new chromophore dyes. The emission and absorption colors of the thiophene-coumarin compounds differed between their solution and film forms. Electrochemically, the highest occupied molecular orbital levels of the coumarins increased with the 3,4-ethylenedioxythiophene group, leading to a narrowing of the band gap, while the phenyl bridge weakened the donor–acceptor interaction, expanding the band gap.

## 1 Introduction

Conjugated polymers (CPs) are structures that offer solution processability, structural tunability, and mechanical flexibility over their inorganic materials, which can be designed to absorb/emit light and conduct electrical currents.^1^ CPs have become critical components of sustainable and low-cost electronic devices such as organic photovoltaics (OPVs),^2^ organic light-emitting diodes (OLEDs),^3^ electro(photo)chromic windows,^4^ and organic field-effect transistors (OFETs).^5^ Therefore, synthesized conjugated polymers have increased dramatically in recent years.^6^ CPs typically consist of peripheral flexible solubilizing side chains and π-conjugated backbones.^7^ Since π-conjugated backbones determine the optoelectronic characteristics of polymers, the majority of research efforts have focused on this area.^8^ The energy band gap between these materials’ valence band [highest occupied molecular orbital (HOMO)] and conduction band [lowest unoccupied molecular orbital (LUMO)] is crucial for understanding their properties. Especially in optoelectronic devices, organic materials with low energy band gaps have a prominent place.^9^ There are numerous approaches for adjusting the HOMO and LUMO energies in materials. The energy band gap can be adjusted by the length of the conjugated backbone, enforcing coplanarity of the rings and multiple bonds through covalent or noncovalent interactions, and increasing the quinoidal character of the backbone by reducing the aromaticity of the monomer units.^10^ However, donor–acceptor polymers, which are created by combining suitable electron-rich donor units with electron-poor acceptor units, are the most preferred method for narrow band gaps.^11^

The precursor routes for conjugated materials used in these applications are crucial to understand. The incorporation of such materials into devices is made easier by processable precursor polymers with adjustable optoelectronic properties.^12^ To date, many types of organic precursor molecules and their polymers have been investigated, such as dibenzophenazine-arylamine derivatives,^13^ polymers and frameworks containing triazine,^14^ polymers using biphenyls in the polythiophene backbone, and others.^15^ Thiophene exhibits weaker aromaticity, less steric hindrance, and additional sulfur–sulfur interactions in the solid state when compared with the other molecules. Therefore, thiophene-based functional materials have been crucial to the progress of organic electronics.^16^ Thiophene sulfur has an oxidation state and two lone pairs of electrons, one of which participates in ring aromatization. Among 3,4-ethylenedioxythiophene (EDOT) and thiophene-based conjugated molecules, especially EDOT-containing molecules, they have high stability, high optical properties, and low oxidation. Because of these advantages, EDOT and thiophene units contribute significantly to the synthesis of D–A–D type-conjugated backbones and their polymers.^17^ Heteroaromatic rings such as thiophene and EDOT are incorporated into coumarin-based dyes and pigments as they act as the building blocks of the π-bridge in the push–pull system, resulting in fluorescent materials that offer distinctive photochemical and photophysical properties.^18^ Due to their strong photoluminescence quantum yields, coumarin and its derivatives are highly sought-after in optoelectronic applications such as organic solar cells, fluorescence probes, OLED, and OFET.^19^ In addition, asymmetric molecules consistently outperform symmetrical molecules among all precursor molecules for optoelectronic devices because they are more likely to sharpen and result in higher carrier transport efficiency.^20^ However, research on polymer donors with asymmetric structures is rare. Additionally, the complicated synthesis process of asymmetric units impedes the development of these molecules.^21^

In this study, we demonstrated the viability of the electroactive monomer group-based “lead polymer” approach using various polymer backbones with new types of symmetrical and asymmetrical structures. Two donors were added to the precursor structures to form D–A–D type chromophores to enable stronger intramolecular charge transfer (CT) and subsequently lower the band gap energy. In general, the combination of thiophene and coumarin in optoelectronic applications shows promise for the development of high-performance and stable electronic devices.

## 2 Experimental Section

### 2.1 Materials and Equipment

The starting materials, 5-bromosalicylaldehyde, *p*-bromophenylacetic acid, thiophene 2-acetonitrile, anhydrous sodium acetate, piperidine, acetic anhydride, thiophene-2-boronic acid pinacol ester, tetrakis(triphenylphosphine)palladium(0), 3,4-ethylenedioxy-2-(tributylstannyl)thiophene, bis(triphenylphosphine)palladium(II) dichloride, and all solvents were purchased from Sigma-Aldrich and TCI.

The progress of the reactions and the purity of the products were checked by the thin-layer chromatography technique. Fourier transform infrared (FT-IR) spectra were recorded on a PerkinElmer Spectrum 100 FT-IR Spectrometer with ATR capability. C, H, and N microanalyses were performed on the LECO, CHNS-932 elemental analyzer. HRMS analyses were performed with the LC-HRMS Thermo Q Exactive device. ^1^H NMR and ^13^C NMR spectra were recorded on a Bruker Avance III 500 spectrometer in deuterated chloroform (CDCl_3_). Electronic absorption spectra (UV–Vis) were recorded on a Shimadzu UV-2450 UV–Visible Spectrophotometer in dichloromethane (DCM). Fluorescence excitation and emission spectra were recorded on a Hitachi F-7000 spectrofluorometer using a 1 cm path length cuvette at room temperature in DCM. The optical band gaps (*E*_g_) of coumarins were calculated from the absorption edges (λ_onset_) using the equation *E*_g_ = 1241/λ_onset_. Thermal gravimetric analysis (TGA) and differential scanning calorimetry (DSC) measurements were carried out using a TA Instruments SDT-Q600 (heating rate of 10 °C min^–1^) and operated under a nitrogen flow (50 mL min^–1^).

To understand the electrochemical properties and calculate the band gap energy levels, cyclic voltammetry (CV) was carried out using a three-electrode cell system comprising a glassy carbon electrode as the working electrode and a platinum wire and saturated calomel electrode as the counter and reference electrodes, respectively. Also, these measurements were recorded at the Dropsens μStat 400 Bipotentiostat/Galvanostat instrument in DCM, containing 0.1 M *n*-Bu_4_NPF_6_ as the supporting electrolyte-recorded at a scan speed of 100 mV s^–1^. The electrochemical HOMO or LUMO position of coumarins was determined from the oxidation–reduction onset potentials and calibrated against the ferrocene redox couple using the equation *E*_HOMO or LUMO_ = −e(*E*_ox_^ons^ or E_red_^ons^ – *E*_Fc_) + (−4.8 eV).^22^ Atomic force microscopy (AFM) images were obtained in both the height and phase-contrast modes using a Digital Instruments Dimension 3000 scanning force microscope in the tapping mode. Transmission electron microscopy (TEM) was performed using a JEOL 2000CX instrument at a 200 kV accelerating voltage.

As an important photophysical parameter, fluorescence quantum yield (Φ_F_) is determined by the comparative method according to eq 1
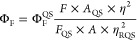
1where *F* and *F*_QS_ are the areas under the fluorescence emission curves of **ETQ**, **EPQ**, **TPQ**, and **DTQ** and the standard, respectively. *A* and *A*_QS_ are the respective absorbances of **ETQ**, **EPQ**, **TPQ**, and **DTQ** and standard at the excitation wavelengths, respectively. *n*^2^ and *n*_QS_^2^ are the refractive indices of DCM used for the sample and standard, respectively. The fluorescence quantum yield of quinine sulfate, which was dissolved in 0.1 M H_2_SO_4_, was determined to be 0.54 in the literature,^23^ and it was used as a standard in this study. The absorbances of the studied **ETQ**, **EPQ**, **TPQ**, **DTQ**, and the standard quinine sulfate were kept at *ca*. 0.05 at the excitation wavelength.

Fluorescence lifetime (τ_F_) was measured in DCM with appropriate exponential calculations by HORIBA-Jobin-Yvon-SPEX Fluorolog 3-2iHR, which has a Fluoro Hub-B Single Photon Counting Controller. The signals were recorded by the time-correlated single-photon counting (TCSPC) module. For that purpose, a NanoLED (390 nm) was used as the excitation source.

Density functional theory (DFT)^24^ was used to investigate molecular structures and molecular properties. The molecular structures of the investigated molecules were optimized by Gaussian16^25^ and visualized by GaussView 6.0.^26^ The B3LYP functionals^27^ were used for DFT with the 6-31G(d,p) basis set. The true minimum nature of optimized structures was verified with all positive frequencies. The TD-DFT calculation method was used in the B3LYP/6-31G(d,p) level of theory to obtain the theoretical UV–vis spectrum and to examine electronic transitions.^28^ Solvent effects were investigated using the polarizable continuum model^29^ in the ground state, as implemented in Gaussian16. The theoretical band gap was calculated using the equation Band gap (*E*_g_) = *E*_HOMO_ – *E*_LUMO_.

### 2.2 Synthesis

#### 2.2.1 6-Bromo-3-(2-thienyl)coumarin (**1**)

A mixture of 5-bromosaliciylaldehyde (3.0 g, 14.92 mmol) and thiophene-2-acetonitrile (1.83 g, 14.92 mmol) dissolved in a mixture of ethanol (15 mL) and piperidine (1 mL) was refluxed for 7 h. The yellow precipitate was filtered and washed with hexane. The resulting construct was iminocoumarin. The yellow precipitate was mixed with dilute HCl in an ice bath for 2 h. The precipitate was filtered and washed with distilled water until neutral.

Yield: 3.85 g (84%). Anal. Calcd for C_13_H_7_BrO_2_S: C, 50.83; H, 2.30; S, 10.44%; found: C, 50.81; H, 2.28; S, 10.42%. FT-IR (ATR), *v*_max_/cm^–1^: 3056 (Aromatic-CH), 2922–2850 (Aliphatic-CH), 1695 (C=O), 1591 (C=C), 801 (Ar–Br). ^1^H NMR (DMSO-*d*_6_, 400 MHz): δ 8.49 (s, 1H, Coumarin-4H), 7.97 (d, 1H, *J* = 6.10 Hz, Ar–H), 7.81 (d, 1H, *J* = 3.02 Hz, Thiophene-H), 7.73–7.70 (m, 2H, Ar–H, Thiophene-H), 7.40 (d, 1H, *J* = 8.60 Hz, Ar–H), 7.18 (t, 1H, Thiophene-H). ^13^C NMR (DMSO-*d*_6_, 100 MHz): δ: 159.1, 151.7, 135.6, 135.3, 134.2, 130.8, 129.9, 128.1, 127.7, 122.1, 121.8, 118.8, 116.9.

#### 2.2.2 3,6-Dithienylcoumarin (DTQ)

A mixture of 6-bromo-3-(2-thienyl)coumarin (**1**) (500 mg, 1.5 mmol) and thiophene-2-boronic acid pinacol ester (410 mg, 1.8 mmol) was dissolved in a mixture of K_2_CO_3_ (aq) (5 mL, 2.0 M) and toluene (50 mL) and stirred for 30 min under an argon atmosphere. After stirring, Pd(PPh_3_)_4_ (5.0 mol %) was added as a catalyst, and the reaction was refluxed for 24 h. After the reaction solvent was evaporated, the crude product was purified by column chromatography on silica gel using a chloroform-hexane solvent mixture (1:1) as an eluent.

Yield: 340 mg (73%). Anal. Calcd for C_17_H_10_O_2_S_2_: C, 65.79; H, 3.25; S, 20.66%; found: C, 65.77; H, 3.23; S, 20.64%. FT-IR (ATR), *v*_max_/cm^–1^: 3078–3045 (Aromatic-CH), 2922–2850 (Aliphatic-CH), 1699 (C=O), 1602–1561 (C=C). ^1^H NMR (DMSO-*d*_6_, 400 MHz): δ 8.60 (s, 1H, Coumarin-4H), 8.02 (d, 1H, *J* = 1.81 Hz, Ar–H), 7.88 (dd, 1H, *J* = 8.59 and *J* = 1.89 Hz, Ar–H), 7.85 (d, 1H, *J* = 3.44 Hz, Thiophene-H), 7.69 (d, 1H, *J* = 4.96 Hz, Thiophene-H), 7.57 (d, 1H, *J* = 4.96 Hz, Thiophene-H), 7.54 (d, 1H, *J* = 3.44 Hz, Thiophene-H), 7.46 (d, 1H, *J* = 8.59 Hz, Ar–H), 7.18 (dd, 1H, *J* = 3.82 Hz, Thiophene-H), 7.14 (dd, 1H, *J* = 3.82 Hz, Thiophene-H). ^13^C NMR (DMSO-*d*_6_, 100 MHz): δ: 159.4, 151.9, 142.3, 136.4, 135.8, 131.1, 129.6, 129.2, 129.1, 128.0, 127.4, 126.8, 125.1, 124.8, 121.6, 120.4, 117.3. HRMS (ESI): Exact mass calcd 310.01, found 311.01877 [M + H]^+^.

#### 2.2.3 6-(2,3-Dihydrothieno[3,4,*b*]dioxinyl)-3-thienylcoumarin (**ETQ**)

A mixture of 6-bromo-3-(2-thienyl)coumarin (**1**) (500 mg, 1.5 mmol) and 3,4-ethylenedioxy-2-(tributylstannyl)thiophene (1.05 g, 1.8 mmol) was dissolved in toluene (20 mL) and stirred for 30 min under an argon atmosphere. After stirring, Pd(PPh_3_)_2_Cl_2_ (5.0 mol %) was added as a catalyst, and the reaction was refluxed for 48 h. After the reaction solvent was evaporated, the crude product was purified by column chromatography on silica gel using a chloroform-hexane solvent mixture (1:1) as an eluent.

Yield: 380 mg (69%). Anal. Calcd for C_19_H_12_O_4_S_2_: C, 61.94; H, 3.25; S, 17.40%; found: C, 61.93; H, 3.24; S, 17.38%. FT-IR (ATR), *v*_max_/cm^–1^: 3097 (Aromatic-CH), 2918–2858 (Aliphatic-CH), 1704 (C=O), 1599–1561 (C=C). ^1^H NMR (DMSO-*d*_6_, 400 MHz): δ 8.77–8.73 (m, 2H, Coumarin-4H, Ar–H), 8.02 (d, 1H, *J* = 7.10 Hz, Ar–H), 7.87 (d, 1H, *J* = 8.45 Hz, Ar–H), 7.69 (d, 1H, *J* = 8.50 Hz, Ar–H), 7.46 (d, 1H, *J* = 8.51 Hz, Ar–H), 7.18 (d, 1H, *J* = 8.35 Hz, Ar–H), 6.67 (d, 1H, *J* = 8.40 Hz, Ar–H), 4.34 (bt, 2H, CH_2_), 4.26 (bt, 2H, CH_2_). ^13^C NMR (DMSO-*d*_6_, 100 MHz): δ: 159.3, 151.0, 142.7, 139.3, 136.5, 135.7, 130.0, 129.5, 128.9, 127.9, 127.3, 124.8, 121.5, 120.1, 116.9, 114.72, 99.0, 65.3, 64.6. HRMS (ESI): Exact mass calcd 368.02, found 369.02451 [M + H]^+^.

#### 2.2.4 6-Bromo-3-(*p*-bromophenyl)coumarin (**2**)

A mixture of 5-bromosalicylaldehyde (1.5 g, 7.4 mmol), *p*-bromophenyl acetic acid (1.6 g, 7.4 mmol), anhydrous NaOAc (0.9 g, 11.1 mmol), and 12 mL of acetic anhydride was heated at 160–170 °C while stirring under an argon atmosphere for 7 h. After the removal of acetic acid by distillation, the resulting solid was dissolved in a 100 mL THF/methanol (3:1) mixture, and then lithium hydroxide (1.0 g, 42 mmol) in 5 mL of water was added to the suspension. About 2 h later, the reaction mixture was poured into 150 mL of ice water and treated with 10% HCl; the precipitate was collected by filtration, washed with water, and dried. The crude product was purified by recrystallization from methanol. Yield: 2.1 g (75%). Anal. Calcd for C_15_H_8_Br_2_O_2_: C, 47.41; H, 2.12%; found: C, 47.40; H, 2.11%. FT-IR (ATR), *v*_max_/cm^–1^: 3048 (Aromatic-CH), 1714 (C=O), 1599 (C=C), 812 (Ar–Br). ^1^H NMR (CDCl_3_, 400 MHz): δ 7.72 (s, 1H, Coumarin-4H), 7.67 (d, 1H, *J* = 2.46 Hz, Ar–H), 7.61 (dd, 1H, *J* = 8.85 and 2.46 Hz, Ar–H), 7.57 (m, 4H, Ar–H), 7.25 (d, 1H, *J* = 8.5 Hz, Ar–H). ^13^C NMR (CDCl_3_, 100 MHz): δ: 159.6, 152.6, 140.0, 134.7, 131.8, 131.2, 127.4, 122.8, 121.8, 118.8, 116.7.

#### 2.2.5 6-Thienyl-3-(*p*-thienylphenyl)coumarin (**TPQ**)

A mixture of 6-bromo-3-(*p*-bromophenyl)coumarin (**2**) (500 mg, 1.3 mmol) and thiophene-2-boronic acid pinacol ester (750 mg, 3.25 mmol) dissolved in a mixture of K_2_CO_3_ (aq) (5 mL, 2.0 M) and toluene (50 mL) was stirred for 30 min under an argon atmosphere. After stirring, Pd(PPh_3_)_4_ (5.0 mol %) was added as a catalyst, and the reaction was refluxed for 24 h. After the reaction solvent was evaporated, the crude product was purified by column chromatography on silica gel using a chloroform-hexane solvent mixture (1:1) as an eluent.

Yield: 300 mg (63%). Anal. Calcd for C_23_H_14_O_2_S_2_: C, 71.48; H, 3.65; S, 16.59%; found: C, 71.46; H, 3.63; S, 16.58%. FT-IR (ATR), *v*_max_/cm^–1^: 3097–3022 (Aromatic-CH), 1703 (C=O), 1610 (C=C). ^1^H NMR (CDCl_3_, 400 MHz): δ 7.87 (s, 1H, Coumarin-4H), 7.67 (m, 4H, Ar–H, Thiophene-H), 7.69 (d, 2H, *J* = 8.05 Hz, Ar–H), 7.38–7.37 (m, 2H, *J* = 8.50 Hz, Ar–H), 7.33–7.31 (m, 3H, Ar–H, Thiophene-H), 7.12–7.09 (m, 2H, Thiophene-H). ^13^C NMR (CDCl_3_, 100 MHz): δ: 160.4, 152.8, 143.7, 142.6, 139.2, 135.1, 133.6, 131.3, 129.3, 129.1, 128.4, 128.3, 126.0, 125.6, 124.7, 123.7, 120.0, 117.0. HRMS (ESI): Exact mass calcd 386.04, found 387.04996 [M + H]^+^.

#### 2.2.6 6-(2,3-Dihydrothieno[3,4,*b*]dioxinyl)-3-(p-2,3-dihydrothieno[3,4,*b*]dioxinyl)phenylcoumarin (**EPQ**)

A mixture of 6-bromo-3-(*p*-bromophenyl)coumarin (**2**) (500 mg, 1.3 mmol) and 3,4-ethylenedioxy-2-(tributylstannyl)thiophene (1.7 g, 3.9 mmol) was dissolved in toluene (20 mL) and stirred for 30 min under an argon atmosphere. After stirring, Pd(PPh_3_)_2_Cl_2_ (5.0 mol %) was added as a catalyst, and the reaction was refluxed for 48 h. After the reaction solvent was evaporated, the crude product was purified by column chromatography on silica gel using a chloroform-hexane solvent mixture (1:1) as an eluent.

Yield: 485 mg (71%). Anal. Calcd for C_27_H_18_O_6_S_2_: C, 64.53; H, 3.61; S, 12.76%; found: C, 64.51; H, 3.60; S, 12.74%. FT-IR (ATR), *v*_max_/cm^–1^: 3100 (Aromatic-CH), 2955–2858 (Aliphatic-CH), 1707 (C=O), 1602–1572 (C=C), ^1^H NMR (CDCl_3_, 400 MHz): δ 7.88 (d, 1H, *J* = 2.00 Hz, Ar–H), 7.84 (s, 1H, Coumarin-4H), 7.83 (dd, 1H, *J* = 8.78 and *J* = 2.39 Hz, Ar–H), 7.78 (d, 2H, *J* = 8.28 Hz, Ar–H), 7.73 (d, 2H, *J* = 8.59 Hz, Ar–H), 7.43 (d, 1H, *J* = 8.59 Hz, Ar–H), 6.34 (s, 1H, Thiophene-H), 6.33 (s, 1H, Thiophene-H), 4.36–4.32 (m, 4H, –CH_2_), 4.28–4.25 (m, 4H, –CH_2_). ^13^C NMR (DMSO-*d*_6_, 100 MHz): δ: 160.1, 151.9, 142.7, 140.6, 139.6, 139.3, 133.6, 133.1, 129.9, 129.6, 127.2, 125.6, 125.3, 120.4, 115.8, 114.8, 99.9, 98.5, 65.4, 64.7. HRMS (ESI): Exact mass calcd 502.05, found 503.06061 [M + H]^+^.

### 2.3 X-ray Data Collection and Structure Refinement of **ETQ**, **DTQ**, **TPQ**, and **EPQ**

Single crystal data sets for **ETQ**, **DTQ**, **TPQ**, and **EPQ** were collected using a Bruker APEX-II CCD with MoKα (λ = 0.71073) radiation. Data indexing, integration, and absorption correction using APEX suite^30^ were done. Crystal structures were solved using SHELXT^31^ and then refined using SHELXL^32^ in the Olex2^33^ program package. Hydrogen atom locations that were bonded to carbon atoms were geometrically optimized with the following HFIX methods in SHELXL: HFIX 23 for the –CH_2_, and HFIX 43 for the CH of the aromatic rings. Their displacement parameters were set to isotropic thermal displacement parameters (*U*_iso_(H) = 1.2 × *U*_eq_ for CH_aromatic_). In **ETQ**, the atoms C1–C2–C3–C4–S1/C20–C21–C22–C23–S2 (in the thiophene ring) and C18A-C19A/C18–C19 (in the -[1,4]dioxine ring) are disordered over two sites with occupancies of 0.23:0.77 and 0.44:0.56, respectively. In **EPQ**, the atoms C53–C54/C53A–C54A, C28–C29/C28A–C29A, C24–25/C24A–C25A, and C1–C2/C1A–C2A are disordered over two sites with occupancies of 0.68:0.32, 0.53:0.47, 0.71:0.29, and 0.79:0.21, respectively. Table 1 exhibits refinement information on all crystallographic data. Additional crystallographic data with CCDC reference numbers 2256774 (**ETQ**), 2256773 (**DTQ**), 2256771 (**TPQ**), and 2256772 (**EPQ**) has been deposited within the Cambridge Crystallographic Data Center via www.ccdc.cam.ac.uk/deposit.

**Table 1:** Photophysical Parameters of Coumarin Derivatives (**DTQ**, **ETQ**, **EPQ**, and **TPQ**) in DCM

	λ_abs_^max^ (nm)	λ_abs_^max^(nm)	Δ_*v*_® (cm^–1^)	Φ_F_ (%)	τ_F_ (ns)
**DTQ**	353	494	8086	51	5.45
**EPQ**	359	505	8053	47	3.63
**ETQ**	364	539	8920	66	3.36
**TPQ**	340	492	9087	36	4.50

## 3 Result and Discussion

**Scheme 1:**
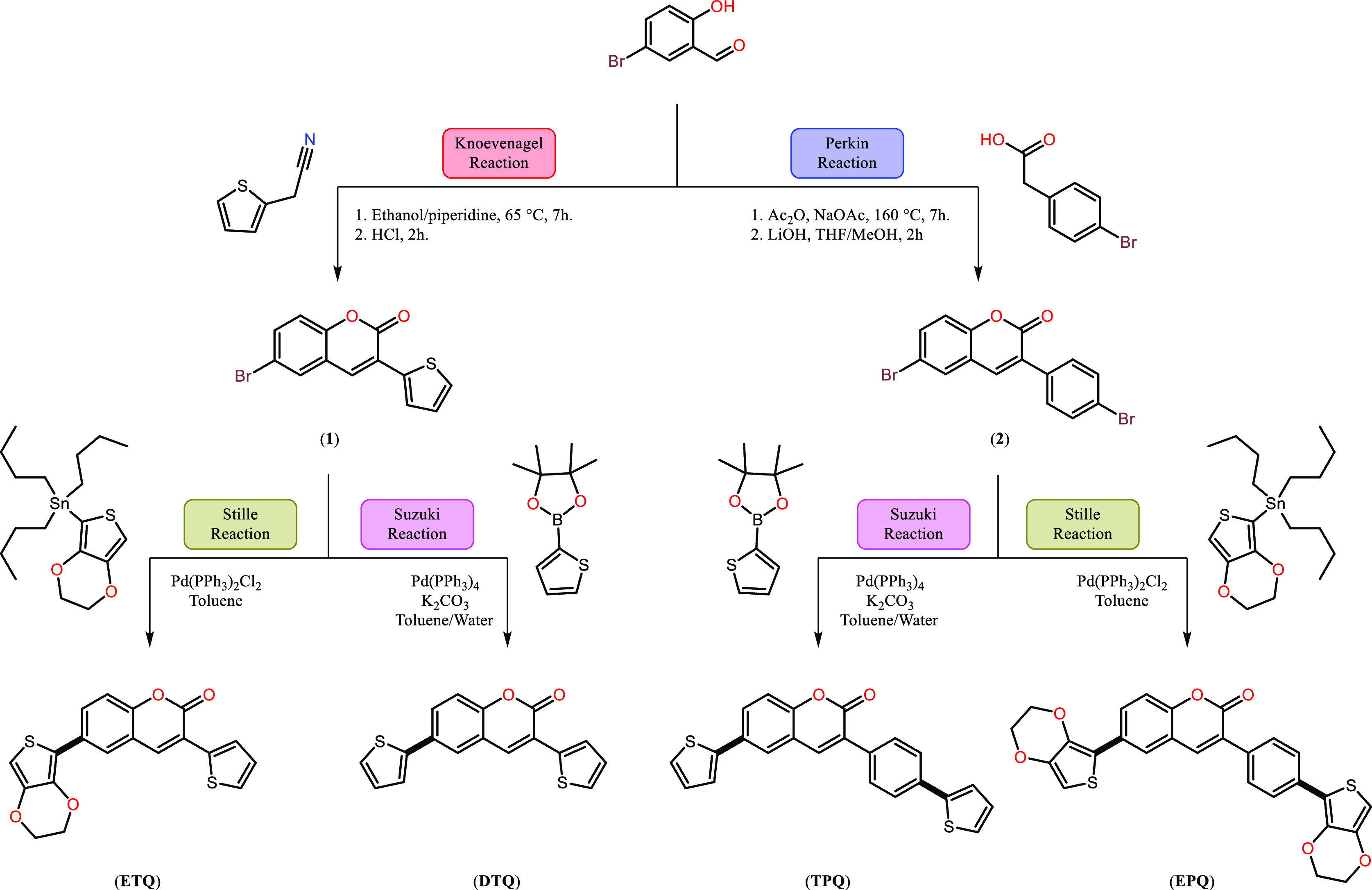
Synthesis Procedure of Thiophene-Coumarin Derivatives

6-Bromo-3-(*p*-bromophenyl)coumarin^34^ and 6-bromo-3-thienyl coumarin^35^ were synthesized according to the literature procedures using the Perkin and Knoevenagel reactions. The target molecules 6-thienyl-3-(*p*-thienylphenyl)coumarin and 3,6-dithienylcoumarin compounds were obtained via the Suzuki–Miyaura, and 6-(2,3-dihydrothieno[3,4,*b*]dioxinyl)-3-(*p*-2,3-dihydrothieno[3,4,*b*]dioxinyl)phenylcoumarin and 6-(2,3-dihydrothieno[3,4,*b*]dioxinyl)-3-thienylcoumarin compounds were synthesized via the Stille coupling reaction using a palladium catalyst (Scheme 1). Synthesis and characterization details (FT-IR, ^1^H NMR, ^13^C NMR, HRMS, TGA, and DSC in Figure S1–S25) of all synthesized molecules are explained in the Supporting Information.

### 3.1 Single-Crystal Structures and Intermolecular Interactions

**Figure 1:**
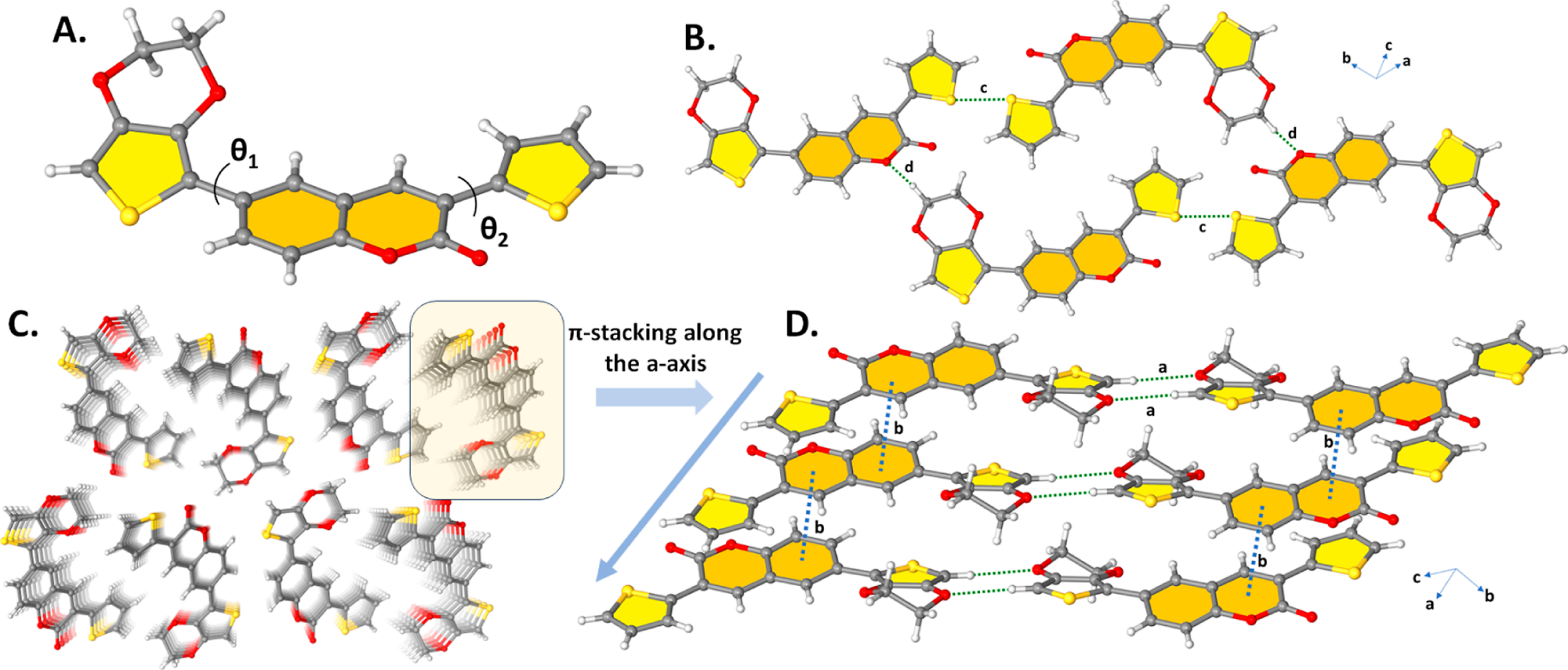
(A) Ball–stick style drawings of the molecular structure showing the corresponding inter-ring dihedral angles (θ1 = 23.19° and θ2 = 6.14°) between the planes in **ETQ** (B) Perspective view of S···S and C–H···O intermolecular hydrogen bonding interactions (C) Perspective view of the molecular arrangement in **ETQ** (D) Intermolecular π···π-stacking interactions and C–H···O intermolecular hydrogen bonding interactions in **ETQ** (*a*: C16–H16···O4, *d*(H···O) = 2.470 Å, *b*: π···π, d(π···π) = 3.553(6) Å, *c*: S1···S1; *d*(S···S) = 3.296(16) Å, *d*: C18–H18C···O2, and *d*(H···O) = 2.594 Å).

**Figure 2:**
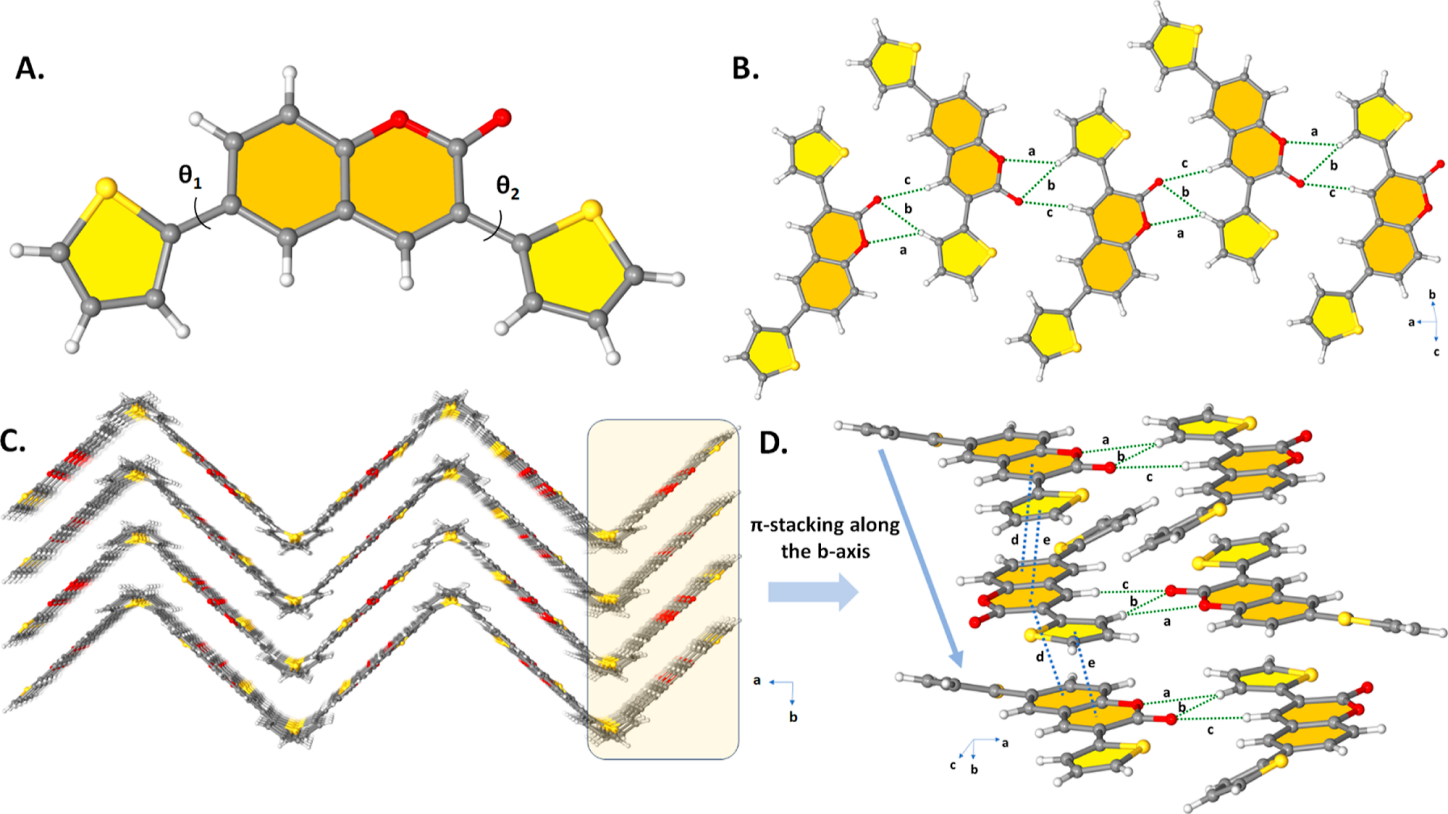
(A) Ball–stick style drawings of the molecular structure showing the corresponding inter-ring dihedral angles (θ1 = 23.48° and θ2 = 4.25°, between the planes in **DTQ** (B) Perspective view of C–H···O intermolecular hydrogen bonding interactions (C) Perspective view of herringbone-like packing viewed down the *c*-axis in **DTQ** (D) Intermolecular π···π-stacking interactions and C–H···O intermolecular hydrogen bonding interactions in **DTQ** (*a*: C3–H3···O2, *d*(H···O) = 2.717 Å, *b*: C3–H3···O1, *d*(H···O) = 2.622 Å, *c*: C7–H7···O1, *d*(H···O) = 2.478 Å, *d*: π···π, *d*(π···π) = 3.608(3) Å, *e*: π···π, and d(π···π) = 3.562(3) Å)).

**Figure 3:**
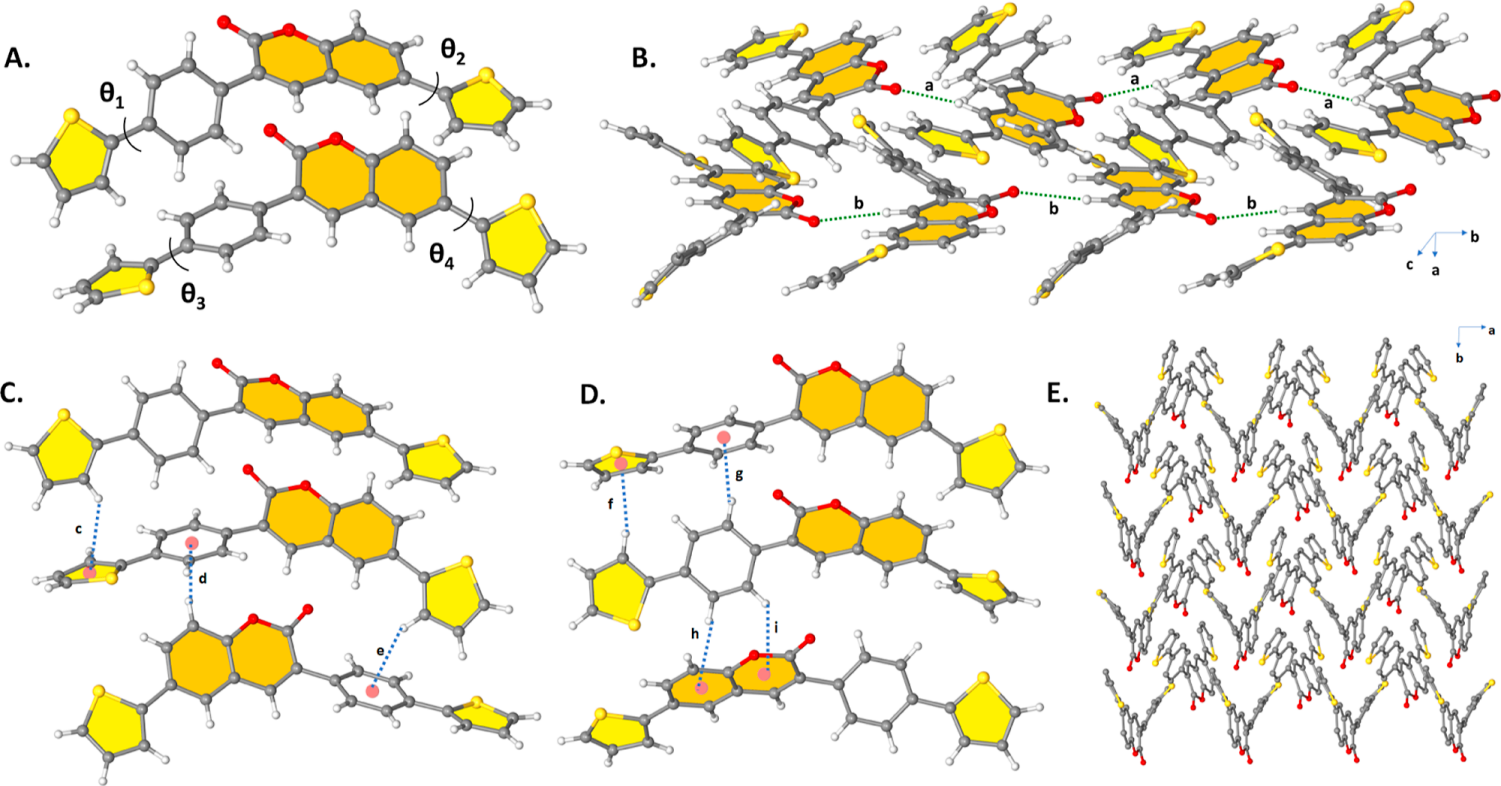
(A) Ball–stick style drawings of the molecular structure showing the corresponding inter-ring dihedral angles (∼θ1 = 51.72°:θ2 = 4.69° and ∼θ3 = 48.61°:θ4 = 26.42°) between the planes in **TPQ**. (B) Perspective view of C–H···O intermolecular hydrogen bonding interactions along the *a*-axis (C,D) Perspective view of intermolecular C–H···π interactions with various distances (*c* = C17–H17···π(thiophene), *d*(H···π) = 2.95 Å, *d* = C3–H3···π(phenyl), *d*(H···π) = 2.83 Å, *e* = C26–H26···π(phenyl), *d*(H···π) = 2.91 Å, *f* = C44–H44···π(thiophene), *d*(H···π) = 2.93 Å, *g* = C38–H38···π(phenyl), *d*(H···π) = 2.91 Å, *h* = C41–H41···π(coumarin), *d*(H···π) = 2.95 Å, *i* = C42–H42···π(coumarin), and *d*(H···π) = 2.93 Å) (E) Herringbone-like arrangement is viewed down the *c*-axis in **TPQ**.

**Figure 4:**
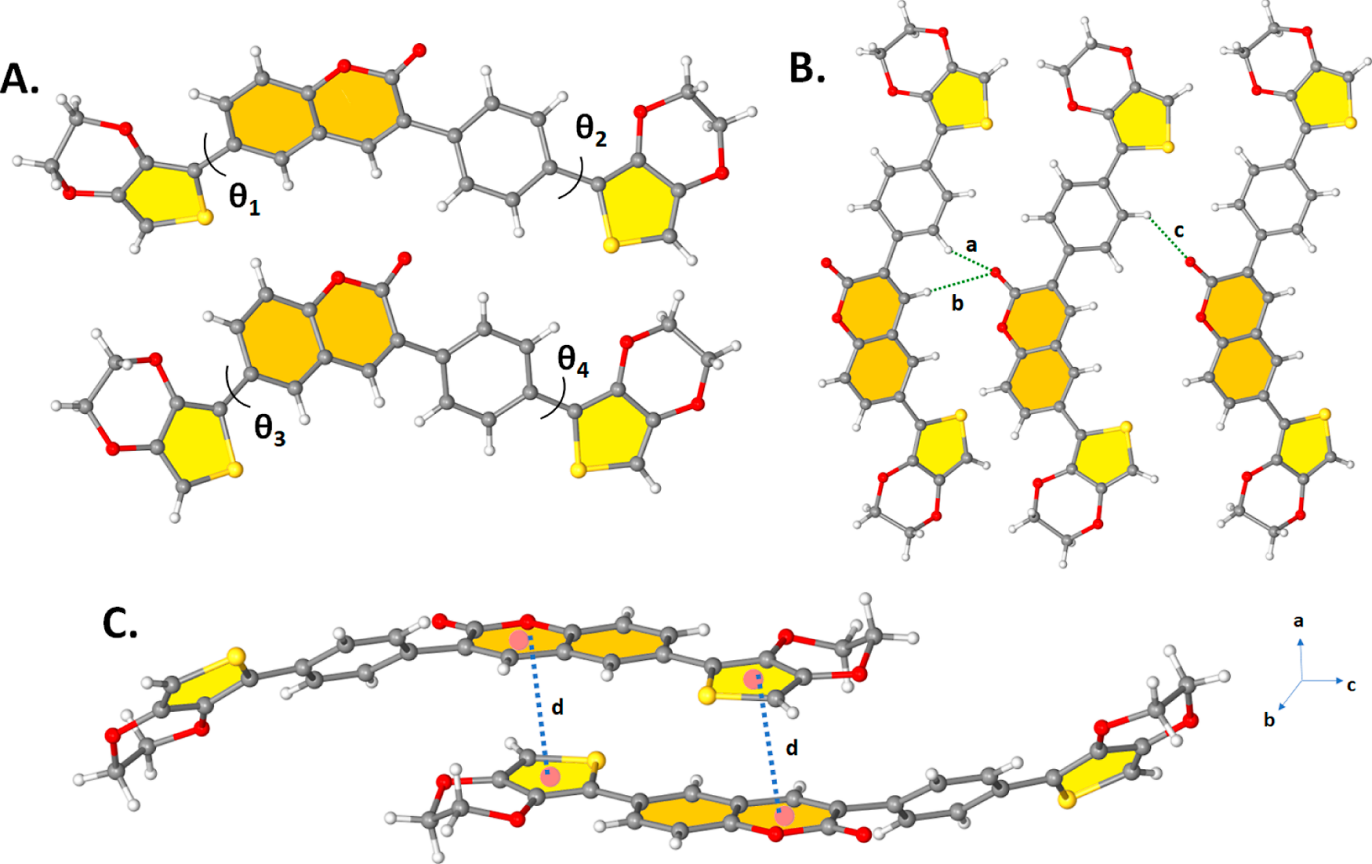
(A) Ball–stick style drawings of the molecular structure showing the corresponding inter-ring dihedral angles (∼θ1 = 5.85°:θ2 = 45.35° and ∼ θ3 = 8.71°:θ4 = 19.13°) between the planes in **EPQ** (B) Perspective view of C–H···O intermolecular hydrogen bonding interactions (*a* = C38–H38···O4, *d*(H···O) = 2.692 Å, *b* = C42–H42···O4, *d*(H···O) = 2.665 Å, *c* = C12–H12···O9, and d(H···O) = 2.614 Å) along the *a*-axis (C) perspective view of the shortest intermolecular π···π interaction (*d* = π(coumarin)···π(thiophene) and *d*(π···π) = 3.574 Å in **EPQ**).

To better understand the molecular structure, packing, and intermolecular interactions in connection with the structure–property relationships of thiophene-coumarin-based organic semiconductors, single-crystal X-ray analysis was used to examine the solid-state features of **ETQ**, **DTQ**, **TPQ**, and **EPQ**. Single crystals of diffraction quality **ETQ**, **DTQ**, **TPQ**, and **EPQ** were produced by diffusing ethanol at room temperature. In **ETQ** and **DTQ**, -dihydrothieno[3,4,*b*]dioxinyl cyanophenyl and five-membered thiophene end units did deviate not too much from the coumarin-backbone, apparently due to the sterically less-encumbered character of the five-membered thiophene ring.^36^ Consequently, our structural approach should provide a substantial advantage for enhancing charge transport in solids. The dihedral angles between dihydrothieno[3,4,*b*]dioxinyl cyanophenyl and thiophene in **ETQ** and **DTQ** are nearly the same and measured to be ∼θ1 = 23.19°:θ2 = 6.14° and ∼θ1 = 23.48°:θ2 = 4.25°, respectively (Figures 1A and 3A). **ETQ** crystallizes in the triclinic space group *P*1̅ showing a π-stacked arrangement along the crystallographic *a*-axis in the solid-state crystal packing (Figure 1C), possibly favoring charge-carrying properties in the solid state. CH···O hydrogen bonding interactions are identified as the dominant intermolecular interactions that govern crystal packing (C16–H16···O4, d(H···O) = 2.470 Å and C18–H18C···O2, d(H···O) = 2.594 Å) and strong π···π staking interactions (d(π···π) = 3.553(6) Å between the coumarin rings along the *a*-axis (Figure 1B,D). In addition, S···S (3.296 Å < *r*_vdw_(S) + *r*_vdw_(S) = 3.60 Å) contacts also exist between the thiophene rings. **DTQ** crystallizes in the orthorhombic space group *Pbca* and exhibits a slipped cofacial π-stacked packing motif with strong interplanar π–π stacking distances of 3.562(3) and 3.608(3) Å through coumarin and the thiophene rings (Figure 2D) along the *b*-axis, again possibly favoring solid-state charge-carrying properties. In **DTQ**, the major intermolecular interactions governing the herringbone motif (Figure 2C) are identified as CH···O hydrogen bonding interactions (C3–H3···O2, d(H···O) = 2.717 Å, C3–H3···O1, d(H···O) = 2.622 Å, C7–H7···O1, and d(H···O) = 2.478 Å) and two strong π···π interactions less than 3.8 Å (*d*(π···π) = 3.562(3) Å and 3.608(3) Å), Figure 2B,D). The asymmetric unit cell of **TPQ** consists of two molecules, each of which is aligned along the *a*-axis by CH···O hydrogen bonding interactions (C9–H9···O1, d(H···O) = 2.637 Å, C34–H34···O3, and d(H···O) = 2.450 Å, Figure 3B). The dihedral angles between coumarin and thiophene in two molecules in the crystal structure of **TPQ** are almost different and measured to be ∼ θ1 = 51.72°:θ2 = 4.69° and ∼ θ3 = 48.61°:θ4 = 26.42°, respectively (Figure 3A). Unlike **ETQ** and **DTQ**, since it has no molecular coplanarity, it was found to prevent efficient cofacial π–π interactions (centroid-to-centroid distance > 4 Å). In this arrangement, the absence of effective π···π interactions led to the predominance of intermolecular C–H···π interactions with distances of 2.83–2.94 Å in the structure (Figure 3C,D). All of these interactions are found to play crucial roles in the solid-state packing of **TPQ** to generate a herringbone-like motif (Figure 3E) with limited π-interactions. Similar to the case for **TPQ**, the asymmetric unit cell of the crystal structure of **EPQ** consists of two molecules. The dihedral angles between coumarin and thiophene rings in **EPQ** are different and measured to be ∼θ1 = 5.85°:θ2 = 45.35° and ∼θ3 = 8.71°:θ4 = 19.13°, respectively (Figure 4A), which shows that the one molecule in the asymmetric unit cell has a nearly planar molecular configuration. **EPQ** molecules are connected to each other by abundant CH···O hydrogen bonding interactions (*a* = C38–H38···O4, d(H···O) = 2.692 Å, *b* = C42–H42···O4, d(H···O) = 2.665 Å, *c* = C12–H12···O9, and d(H···O) = 2.614 Å, Figure 4B) and strong interplanar π–π stacking interactions of 3.574–3.717 Å (less than 3.8 Å) through coumarin and the thiophene rings along the *a*-axis. The shortest π–π contact is displayed in Figure 4C.

### 3.2 Photophysical Properties

Ground-state electronic absorption measurements of thiophene-coumarin derivatives were performed in DCM. To examine the electronic behavior of molecules in solvents of different polarities, measurements were also performed in other solvents. UV–vis measurements were made at a concentration of 1 × 10^–5^ M.

**Figure 5:**
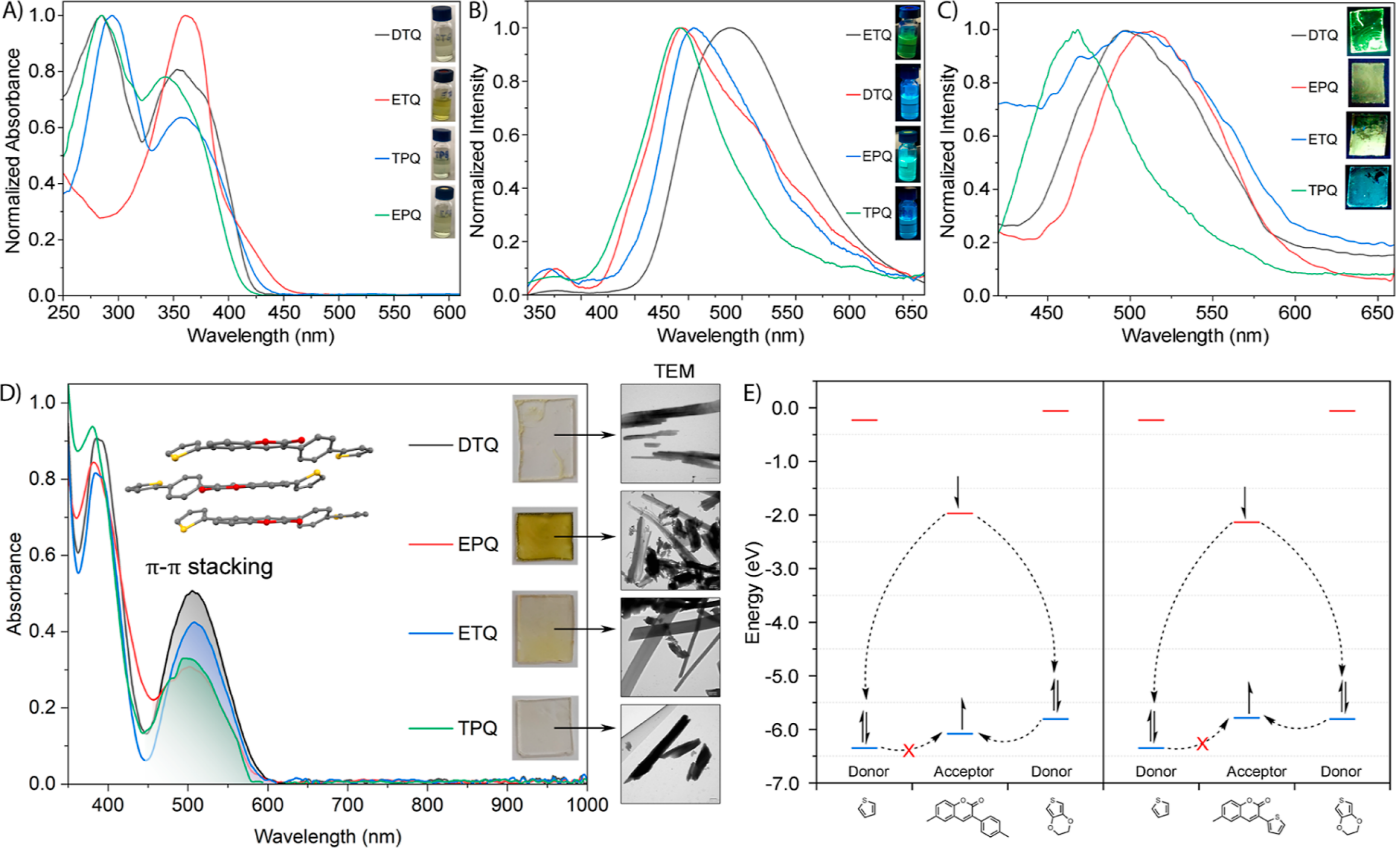
(A) UV–vis spectra of thiophene-coumarin derivatives (**DTQ**, **ETQ**, **EPQ**, and **TPQ**) in DCM. (B) Emission spectra of thiophene-coumarin derivatives (**DTQ**, **ETQ**, **EPQ** and **TPQ**) in DCM (λ_start_^emission^ for **DTQ** = 357 nm; λ_start_^emission^ for **EPQ** = 337 nm; λ_start_^emission^ for **ETQ** = 350 nm; and λ_start_^emission^ for **TPQ** = 347 nm). (C) Emission spectra of thiophene-coumarins (**EPQ**, **TPQ**, **ETQ**, and **DTQ**) on thin films. (D) UV–vis spectra and TEM-AFM images of thiophene-coumarins (**EPQ**, **TPQ**, **ETQ**, and **DTQ**) on thin films.

Pristine 6-bromo-3-(*p*-bromophenyl)coumarin (**1**) absorbed light at 339 nm in DCM, and 6-bromo-3-thienylcoumarin (**2**) absorbed light at 364 nm. While **DTQ**, **EPQ**, and **TPQ** have similar absorption bands, **ETQ** has different absorption bands from the other three compounds. In **DTQ**, **EPQ**, and **TPQ**, two electronic transitions (π···π* transitions) are observed in the 250–450 nm range, with the second electronic transition having a higher absorbance. **ETQ** has a single electronic transition in the 300–400 nm range and a sharp absorption band. The absorption peaks in DCM are 340 nm for **TPQ**, 353 nm for **DTQ**, 359 nm for EPQ, and 364 nm for **ETQ**. When donor thiophene and EDOT groups were added to the coumarin structure, as in 6-bromo-3-thienylcoumarin (**2**), the absorption bands of **TPQ** and **EPQ** were shifted to red. In the case of 6-bromo-3-(*p*-bromophenyl)coumarin (**1**), the absorption of **DTQ** shifted to blue, while the absorption of **ETQ** did not change. No major changes were observed in the absorption of thiophene-coumarin derivatives in different solvents, except for slight peak shifts according to the solvent polarity. In the prepared thin films of thiophene-coumarin compounds, intense red shifts were observed, and a new band in the range of 450–600 nm emerged due to the π···π interactions between their crystal forms. Similar results were reported in the literature.^37^ The absorption peaks of the thin films are 386 and 506 nm for **DTQ**, 382 and 503 nm for **EPQ**, 384 and 508 nm for **ETQ**, and 381 and 494 nm for **TPQ**. The π···π interactions between the molecules were confirmed through both single-crystal and TEM-AFM analyses. The presence of well-organized arrays in the TEM images proved to be a good indicator of well-structured crystals. In AFM, the morphology of the films resulted in smooth surfaces due to the crystal packing, but some agglomerations were observed in certain areas (Figure 5). All UV–vis measurement parameters for thiophene-coumarin compounds are given in Table 1.

The excited-state emission measurements of thiophene-coumarins **ETQ**, **TPQ**, **DTQ**, and **EPQ** were conducted in DCM, and all fluorescence measurements were carried out at a concentration of 1 × 10^–6^ M. The initial compounds (**1** and **2**) exhibit low emission intensities due to the electron-withdrawing effect of the heavy bromine atom. The emission peak wavelength of bromo-thiophene-coumarin (**1**) is 436 nm. By substituting the donor thiophene and EDOT groups into the thiophene-coumarin structure, the emission peak wavelengths shifted to 494 nm for **DTQ** and 539 nm for **ETQ**. The dibromo-phenylcoumarin compound (**2**) shows an emission peak wavelength of 411 nm. Substituting the donor bis-thiophene and EDOT groups into the phenyl-coumarin structure led to emission peak shifts to 505 nm for **EPQ** and 492 nm for **TPQ**. Due to the stronger electron-donating nature of the EDOT group compared to thiophene, the absorption is significantly red-shifted. The prepared thin films of the thiophene-coumarin compounds also exhibited intense emission. While **DTQ** and **EPQ** thin films showed a slight red shift at their emission peak wavelengths, **ETQ** and **TPQ** thin films displayed intense blue shifts in emission, attributed to the alignment of the film surface. The emission peak wavelengths on the film surface are 496 nm for **DTQ**, 507 nm for **EPQ**, 494 nm for **ETQ**, and 468 nm for **TPQ**, and the emission colors of the films differ from those of their solution forms (Figure 5).

Fluorescence quantum yield (Φ_F_) is a measure of the efficiency of photon emission for fluorophores. Thus, Φ_F_ measurements of thiophene-coumarins (**DTQ**, **ETQ**, **EPQ**, and **TPQ**) were calculated in DCM. According to the results, the quantum yields changed with the addition of a phenyl bridge to the coumarin structure and according to the donor substituents (Table 1). Overall, high quantum yields ranging from 65% (**ETQ**) to 75% (**EPQ**) were observed in DCM. Phenyl bridge increased fluorescence quantum yield in coumarins containing strong donor EDOT subgroups (**ETQ** and **EPQ**) and decreased in coumarins containing less donor thiophene subgroups (**TPQ** and **DTQ**). While the phenyl group increased the CT and contributed strongly to the donor–acceptor (D–A) effect with EDOT, it decreased the CT with thiophene and made a weaker contribution to the D–A effect.

**Figure 6:**
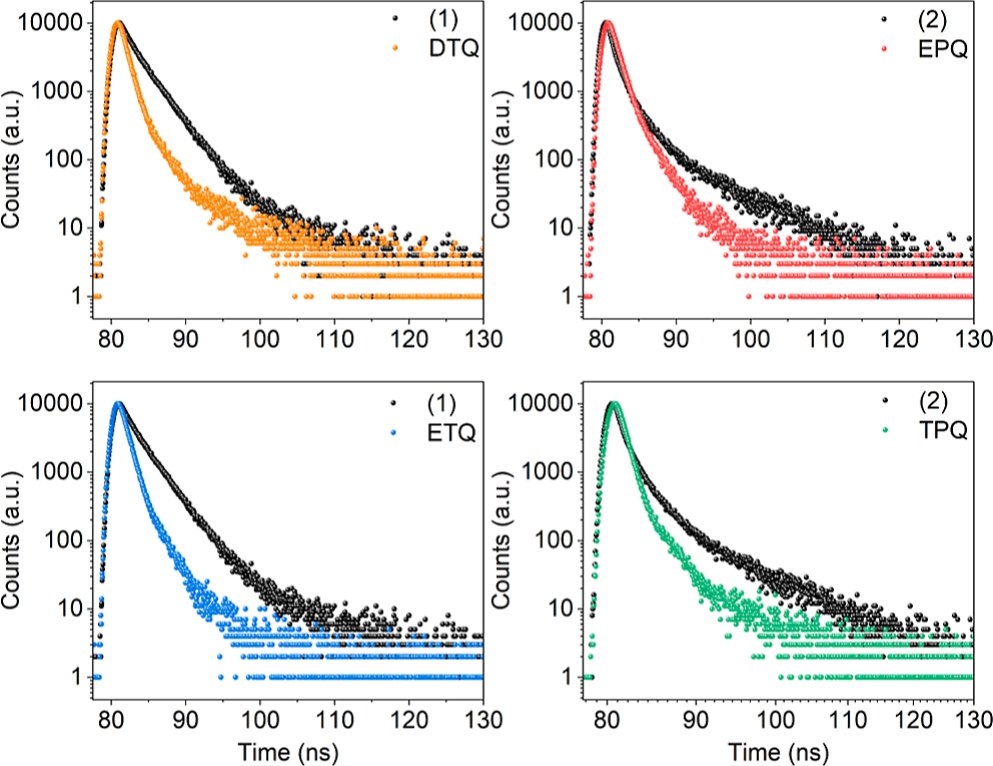
TCSPC trace for **DTQ**, **EPQ**, **ETQ**, and **TPQ** in DCM. (λ^em^ for **DTQ** = 490 nm; λ^em^ for **EPQ** = 515 nm; λ^em^ for **ETQ** = 550 nm; and λ^em^ for **TPQ** = 500 nm).

The excited-state lifetimes (τ_F_) of pristine coumarins (**1**, **2**) and coumarin-thiophenes (**EPQ**, **TPQ**, **ETQ**, and **DTQ**) were measured in DCM using the time-dependent single-photon counting method (Figure 6). The donor thiophene groups increased the fluorescence lifetime of the coumarins, while the EDOT groups decreased the fluorescence lifetime because the EDOT moiety is a stronger donor group than the thiophene moiety and affects the electron distribution on coumarin more, thus the fluorescence lifetime is longer. The longest fluorescence lifetime belongs to the pristine bromo-thiophene-coumarin (**1**), with a lifetime of 5.45 ns. When thiophene and EDOT groups are added to this compound, the fluorescence lifetimes decrease to 3.36 ns for **ETQ** and 5.45 ns for **DTQ**. The fluorescence quantum yield for phenyl-bridged dibromocoumarin (**2**) was 2.74 ns. The fluorescence quantum yields were relatively unchanged with the substitution of the thiophene and EDOT groups, with values of 3.63 ns for EDOT-linked coumarin (**EPQ**) and 4.50 ns for thiophene-linked coumarin (**TPQ**). The electrochemical and theoretical band gaps of thiophene derivatives (**DTQ** and **TPQ**) are wider, while those of EDOT derivatives (**EPQ** and **ETQ**) are narrower. In the excited state, electrons tend to relax by emitting more photons in the wide band gap. Therefore, the fluorescence lifetime values are in positive correlation with the band gap values.

### 3.3 Electrochemistry

**Figure 7:**
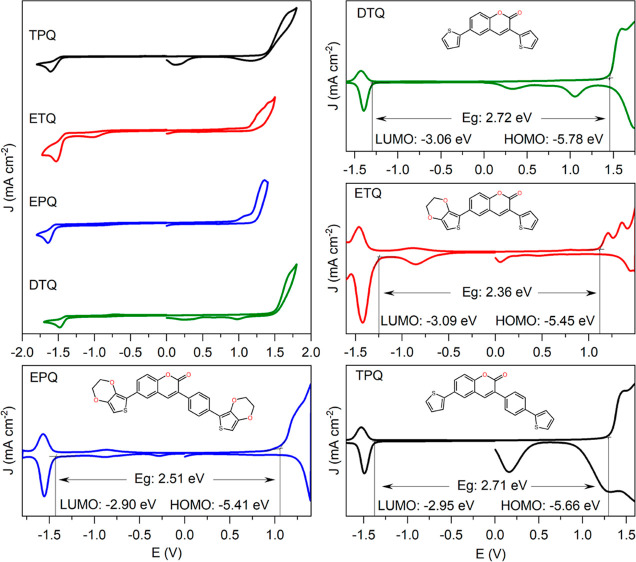
CV and DPV voltammograms of thiophene-coumarin derivatives in a 0.1 M TBAPF_6_/DCM electrolyte solution at a scan rate of 100 mV/s Ag wire.

Electrochemical characterization of thiophene-coumarin derivatives (**DTQ**, **EPQ**, **ETQ**, and **TPQ**) was carried out via CV and differential pulse voltammetry (DPV) techniques in a 0.1 M TBAPF6/DCM electrolyte solution (Figure 7).

When the anodic scan is examined, it is observed that the oxidation potential decreases gradually, and accordingly, the HOMO level increases as a result of the progressive modification of the conjugated main chain through the gradual substitution of the thiophene group with the EDOT group. On the other hand, upon cathodic scanning, a single quasi-reversible reduction peak is observed, which belongs to the coumarin acceptor group. Here, it is observed that the phenyl bridge between the coumarin acceptor and the thiophene or ethylenedioxythiophene donor weakens the donor–acceptor interaction, leading to an increase in electron density on the coumarin acceptor, thereby slightly elevating the reduction potential and shifting the LUMO position upward. Accordingly, the HOMO–LUMO band gap values are calculated as 2.36 eV for **ETQ**, 2.51 eV for **EPQ**, 2.71 eV for **TPQ**, and 2.72 eV for **DTQ**. These data comply with each other when compared with the optical and theoretical band gaps. As a result, the HOMO and LUMO positions in the synthesized structures were easily adjusted through minor modifications occurring in the structure.

### 3.4 DFT Study

The molecular geometry optimizations of thiophene-coumarin compounds (**DTQ**, **EPQ**, **ETQ**, and **TPQ**) were calculated by using the DFT method, and their molecular structures were obtained from crystal information files (CIF) before the calculation.

Compared to the electronic energies of molecular systems, the structure with the lowest energy is the one in which the bithiophene group is directly attached to the coumarin ring (**DTQ**). The highest energy structure is that with two attached EDOT groups (**EPQ**). The energies of the **ETQ** and **TPQ** structures are quite similar to each other. When evaluated for dipole moments, the most polar structure is **EPQ**, while the compound with the lowest polarity is the symmetric structure **DTQ** (Table 2).

**Table 2:** Chemical Data of Thiophene-Coumarin Derivatives (**DTQ**, **EPQ**, **ETQ**, and **TPQ**) Calculated by DFT

	compounds			
chemical properties	DTQ	EPQ	ETQ	TPQ
electronic energy (E)	–43556.38	–62243.63	–49756.24	–49843.90
dipole moment (μ)	3.51	6.64	4.85	3.87
LUMO + 1 (*E*_LUMO+1_)	–6.08	–5.56	–5.81	–5.97
LUMO (*E*_LUMO_)	–5.72	–5.27	–5.52	–5.65
HOMO (*E*_HOMO_)	–2.21	–1.88	–2.07	–2.11
HOMO – 1 (*E*_HOMO–1_)	–1.27	–1.09	–1.17	–1.25
band gap (*E*_GAP_)	3.51	3.39	3.45	3.54

**Figure 8:**
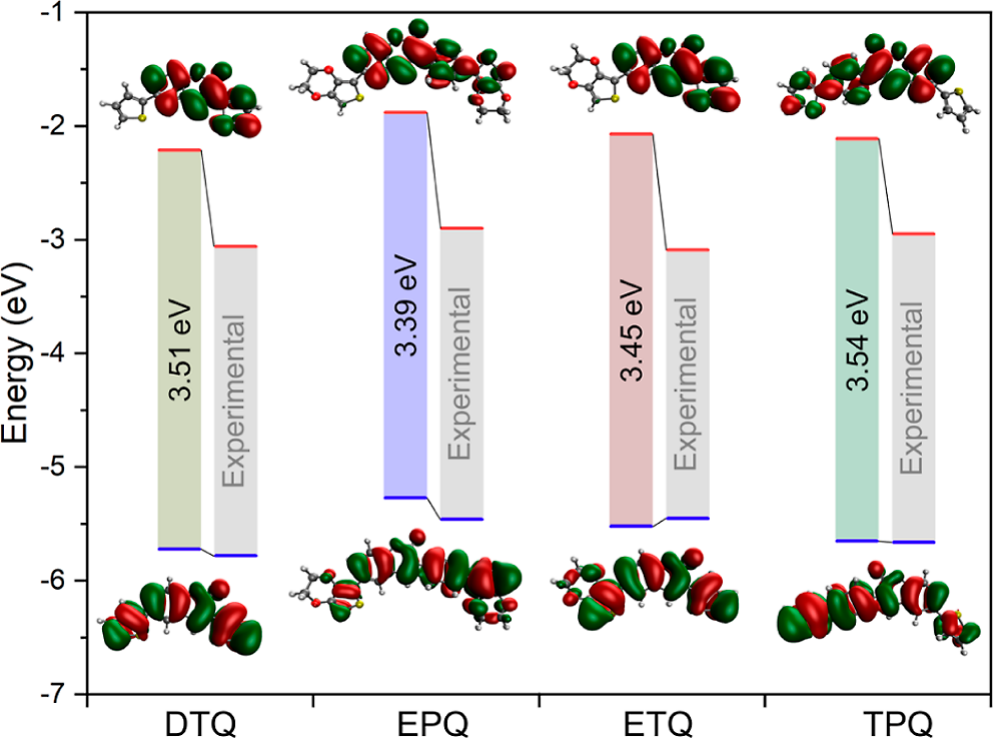
DFT-calculated energy levels and band gaps of thiophene-coumarin derivatives compared with experimental values.

The calculated molecular orbitals are correlated to experimental data. The HOMO energy levels are quite similar, while the LUMO values are slightly lower in energy. The calculated band gaps are 3.51 eV for **DTQ** (experimental 2.72 eV), 3.39 eV for **EPQ** (experimental 2.51 eV), 3.45 eV for **ETQ** (experimental 2.36 eV), and 3.45 eV for **TPQ** (experimental 2.71 eV). The electron clouds of HOMO are located throughout the structures of all four compounds. The LUMO orbitals are located throughout the structure of **DTQ**, **EPQ**, and **ETQ**, except for the substituents attached to the C-6 carbon of coumarin. In **TPQ**, on the other hand, the LUMO orbitals are located throughout the structure, except for the para-thiophene group in the phenyl ring attached to the C-3 carbon of coumarin (Figure 8).

**Figure 9:**
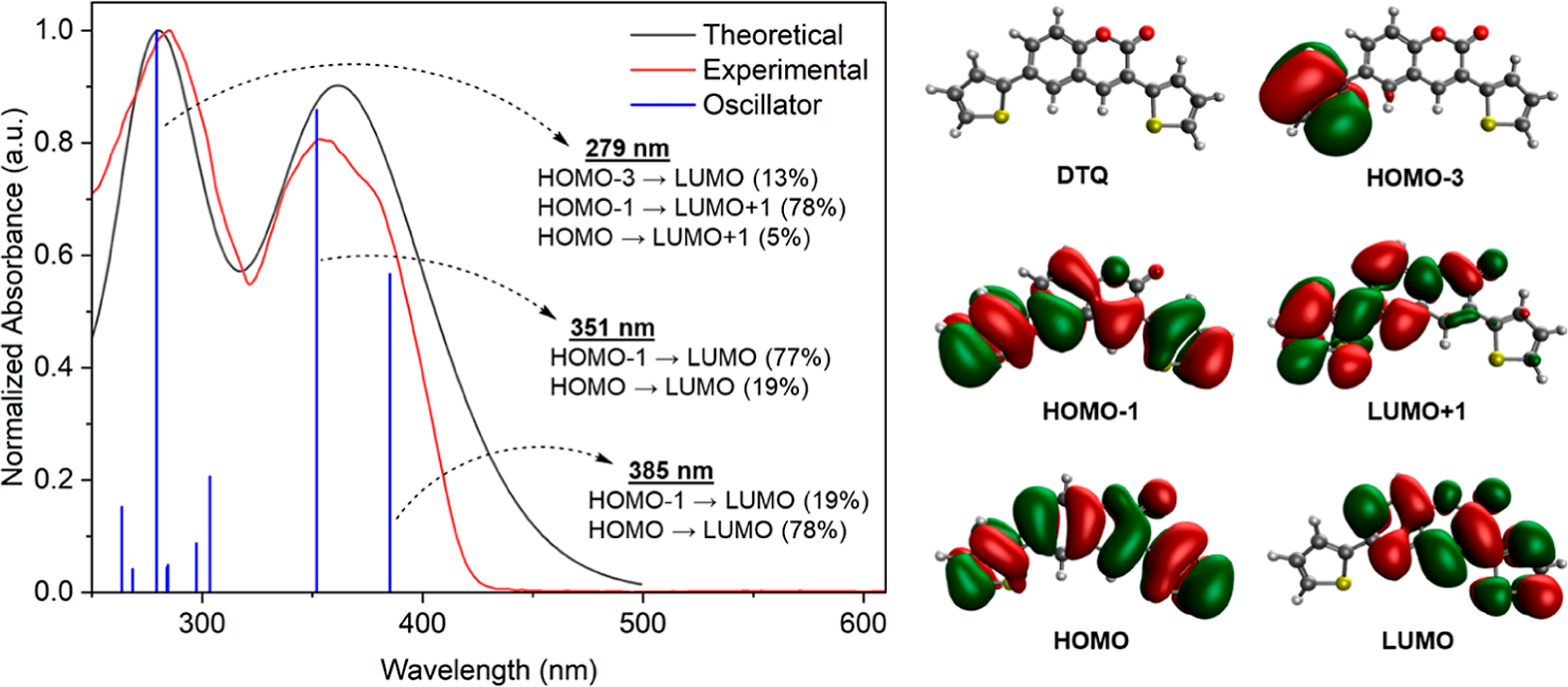
DFT-calculated absorption spectrum of **DTQ** compared with the experimental spectrum in DCM.

The UV–vis absorption spectrum of the compound **DTQ** calculated by DFT shows three electronic transitions. The experimental main absorption peak is at 357 nm, while the theoretical absorption peak is at 354 nm, and they overlap closely. The second absorption peak is at 285 nm for the experimental and 280 nm for the calculated. According to the oscillator strengths, the main peak occurs at 354 nm and is characterized by 77% major contributions from HOMO – 1 to LUMO and 19% major contributions from HOMO to LUMO. In the second peak, major contributions are from HOMO – 3 to LUMO (13%) and from HOMO – 1 to LUMO + 1 (78%), and the minor contribution is from HOMO to LUMO + 1 (5%) (Figure 9).

The transition at 385 nm, on the other hand, has the opposite contributions, with 19% major contributions from HOMO – 1 to LUMO and 78% major contributions from HOMO to LUMO. The calculated absorption peaks for **TPQ** are 289 and 390 nm. The calculated absorption peaks for the **EPQ** are 302 and 408 nm. The calculated absorption peak for **ETQ** is 354 nm.

**Figure 10:**
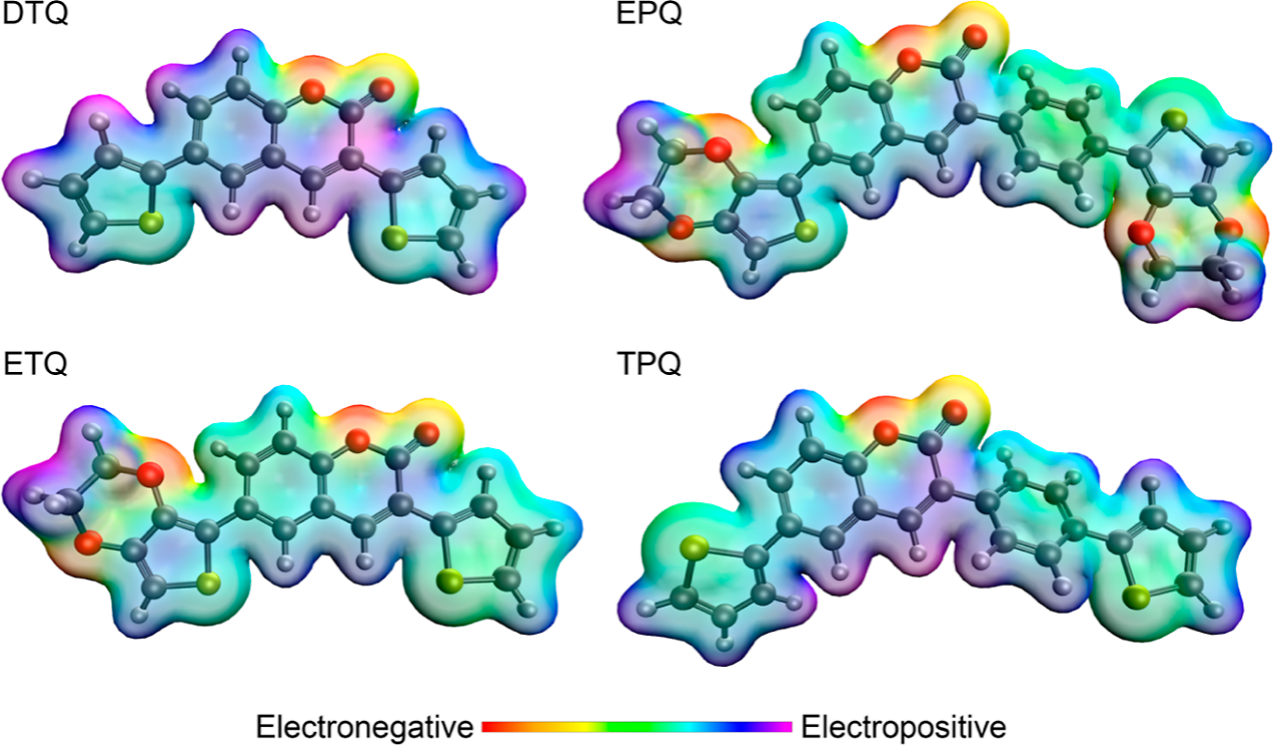
Electron density maps of thiophene-coumarin derivatives (**DTQ**, **EPQ**, **ETQ**, and **TPQ**). Red areas represent electronegativity, and purple areas represent electropositivity.

According to the electron density maps, the carbonyl, the ether bridge of coumarin, and the two ether bridges on EDOT are electron-rich due to the unpaired electrons on the oxygen atoms. Although not as electronegative as oxygen, the sulfur atoms on thiophenes are also electronegative. As a result of these groups attracting electrons toward themselves, the terminal hydrogen atoms on thiophene, EDOT, and coumarin become electronegative, or acidic. The change in electron density is most evident in the **DTQ** structure. In the **EPQ** and **ETQ** structures, the terminal hydrogen atoms of the EDOT subgroup are quite electropositive (Figure 10).

Based on all these results, DFT calculations have predicted the physical, chemical, and optical properties of thiophene-coumarin derivatives, and their compatibility with experimental data has provided an approach for their use as organic semiconductors in optoelectronic applications.

## 4 Conclusions

Four new symmetric and asymmetric thiophene-coumarin units, which can be used in optoelectronic applications, were designed and synthesized through Pd-catalyzed Suzuki and Stille Cross-Coupling reactions. The spectroscopic, optical, electrochemical, computational, and surface properties of the synthesized compounds were investigated. π···π interactions were observed in the crystal packing of thiophene-coumarin derivatives, and these interactions were supported by UV–vis, TEM, and AFM analyses. The photophysical and electrochemical properties of coumarins were explored, and their optical and electrochemical band gaps were found to be in accordance with the theoretical band gaps and HOMO–LUMO energy levels. Coumarin derivatives have strong emissions and exhibit high fluorescence quantum yields and long fluorescence lifetimes. Mega Stokes shifts were observed due to the influence of donor groups. Electrochemically, the HOMO levels of coumarins were raised by the EDOT group, leading to a narrowing of the band gap, while the phenyl bridge weakened the donor–acceptor interaction, resulting in an expansion of the band gap. Coumarin compounds carrying both donor EDOT and thiophene groups simultaneously could be considered as more ideal candidates for semiconductors, and the data in this study can be used as a steppingstone.

## Supporting Information Available

FT-IR spectra, NMR spectra, and optical properties (PDF)
