# Supplementary material for: Symmetrical and Asymmetrical Thiophene-Coumarin-Based Organic Semiconductors
Source: ACS Omega. 2023 Dec 8;9(3):3305–16. doi: 10.1021/acsomega.3c05602 (PMC10809254; doi:10.1021/acsomega.3c05602)
Supplement: Supplementary file 1 — ao3c05602_si_001.pdf [file ao3c05602_si_001.pdf]

**SUPPORTING INFORMATION**

# Symmetrical and asymmetrical thiophene-coumarin-based organic semiconductors

Sinem Altınışık,[a] Mücahit Özdemir,[b] Arzu Kortun,[a] Yunus Zorlu,[c] Bahattin Yalçın,[b] Baybars Köksoy,[d] Sermet Koyuncu[a]*

[a]*Çanakkale Onsekiz Mart University, Department of Chemical Engineering, 17020 Çanakkale, Türkiye.*

[b]*Marmara University, Department of Chemistry, 34722 İstanbul, Türkiye.*

[c]*Gebze Technical University, Department of Chemistry, 41400 Kocaeli, Türkiye.*

[d]*Bursa Technical University, Department of Chemistry, 16310 Bursa, Türkiye.*

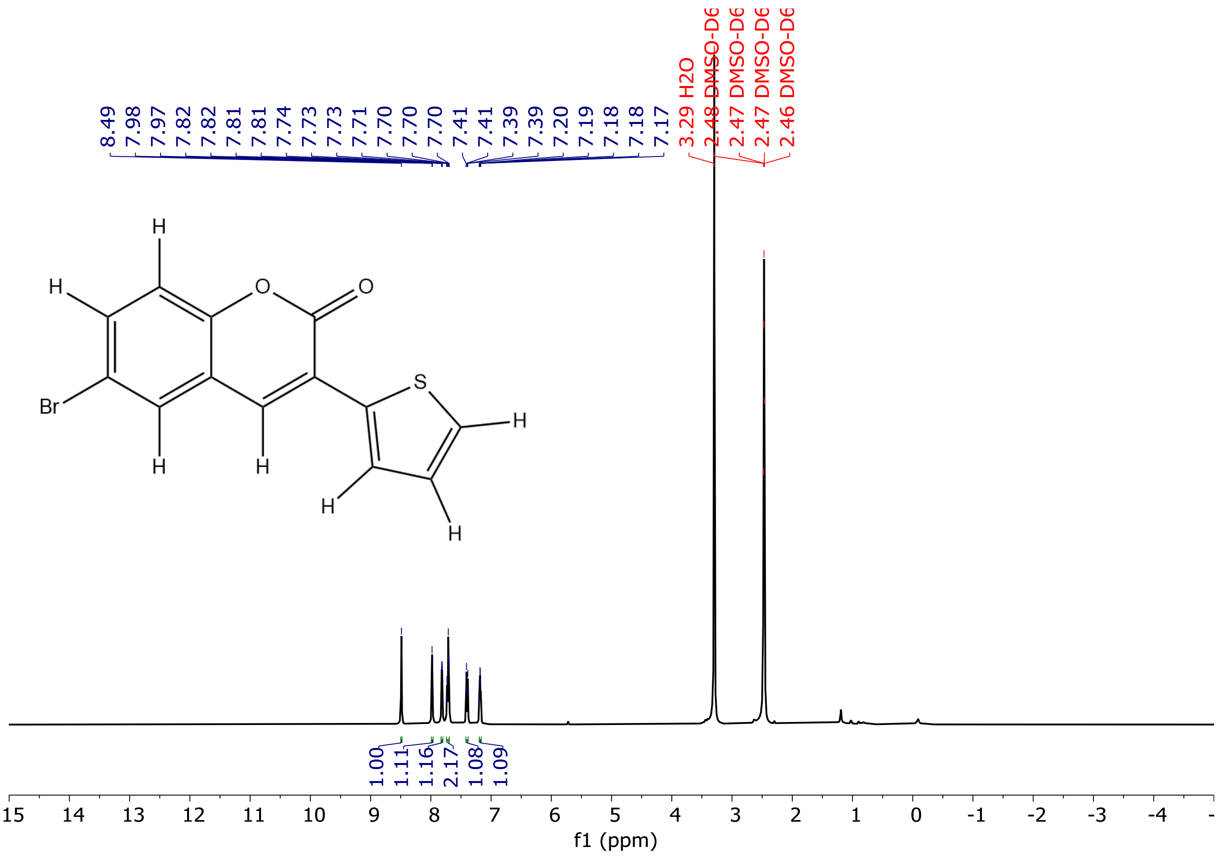

**Figure S1.** $^{1}$H-NMR spectrum of 6-bromo-3-(2-thienyl)coumarin (**1**).

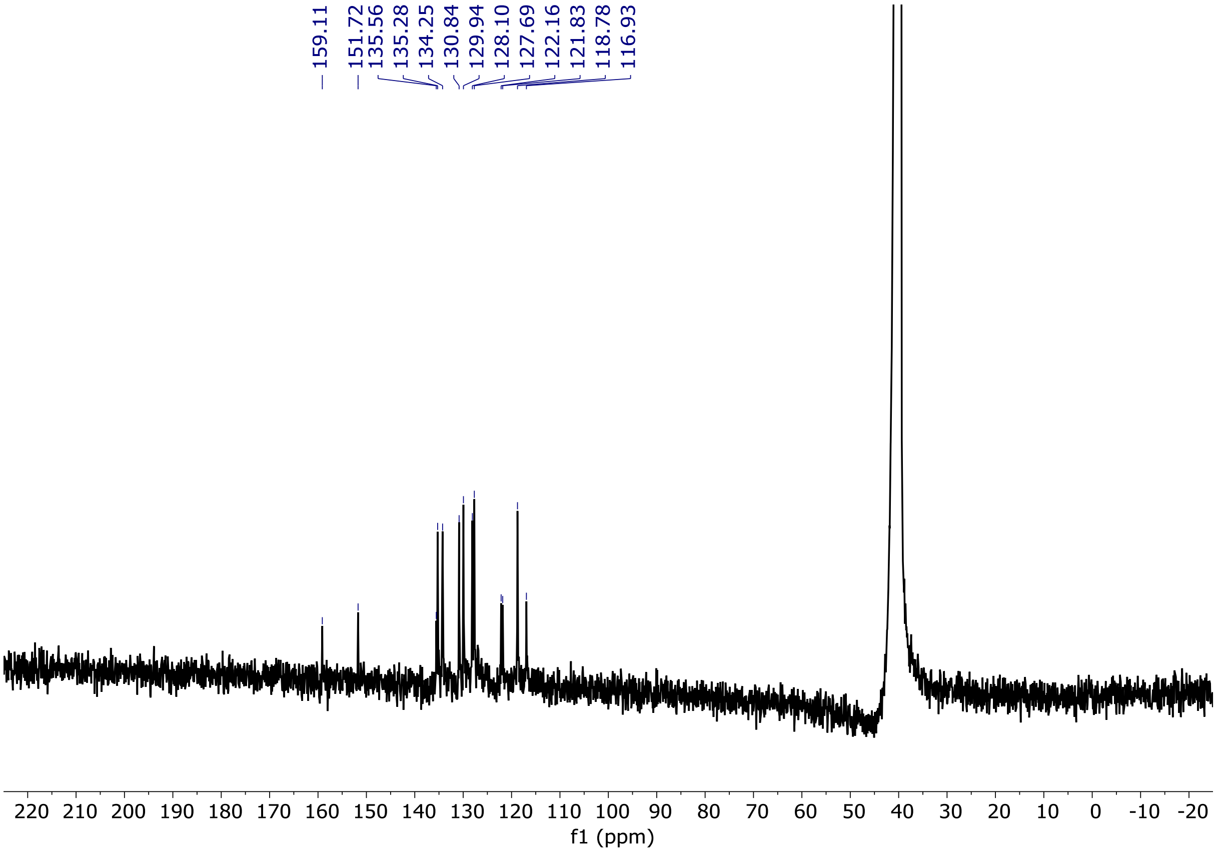


**Figure S2.** $^{13}$C-NMR spectrum of 6-bromo-3-(2-thienyl)coumarin (**1**).

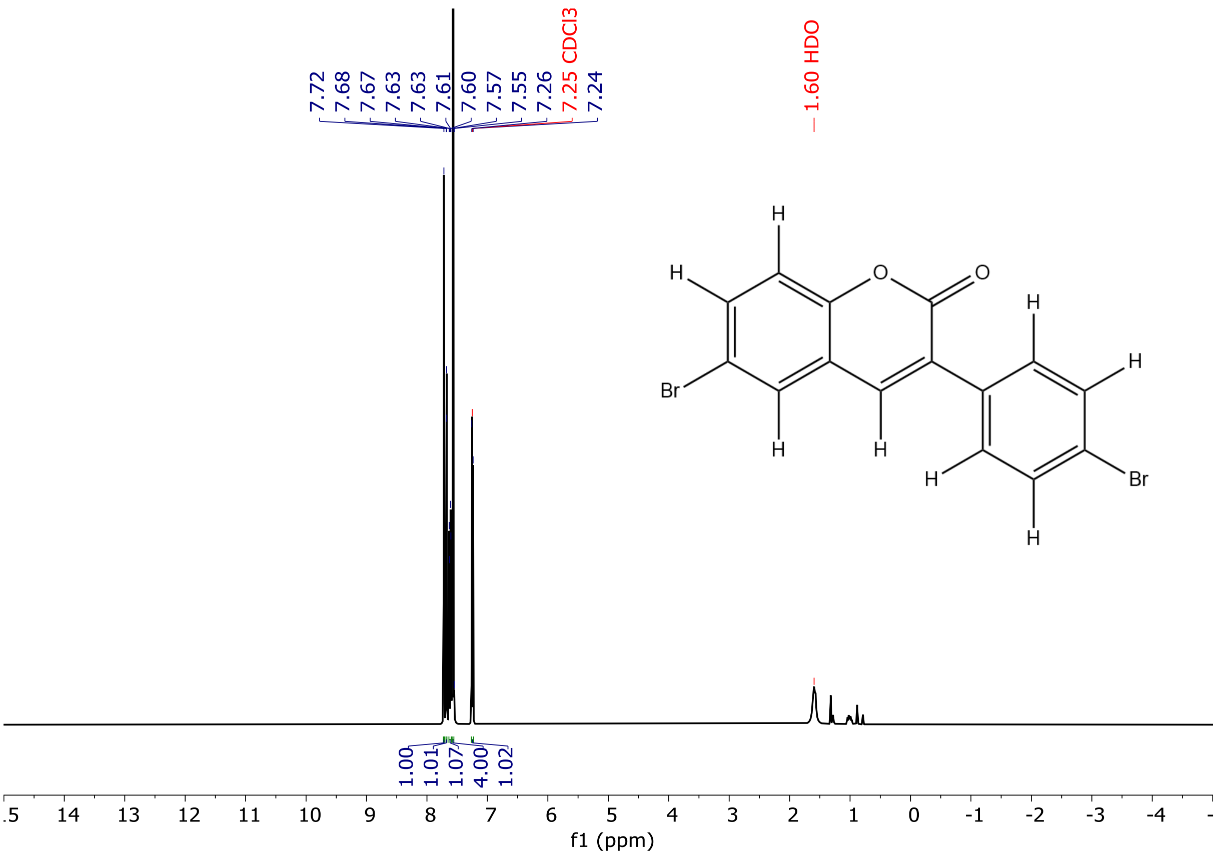


**Figure S3.** $^1$H-NMR spectrum of 6-bromo-3-(*p*-bromophenyl)coumarin (**2**).

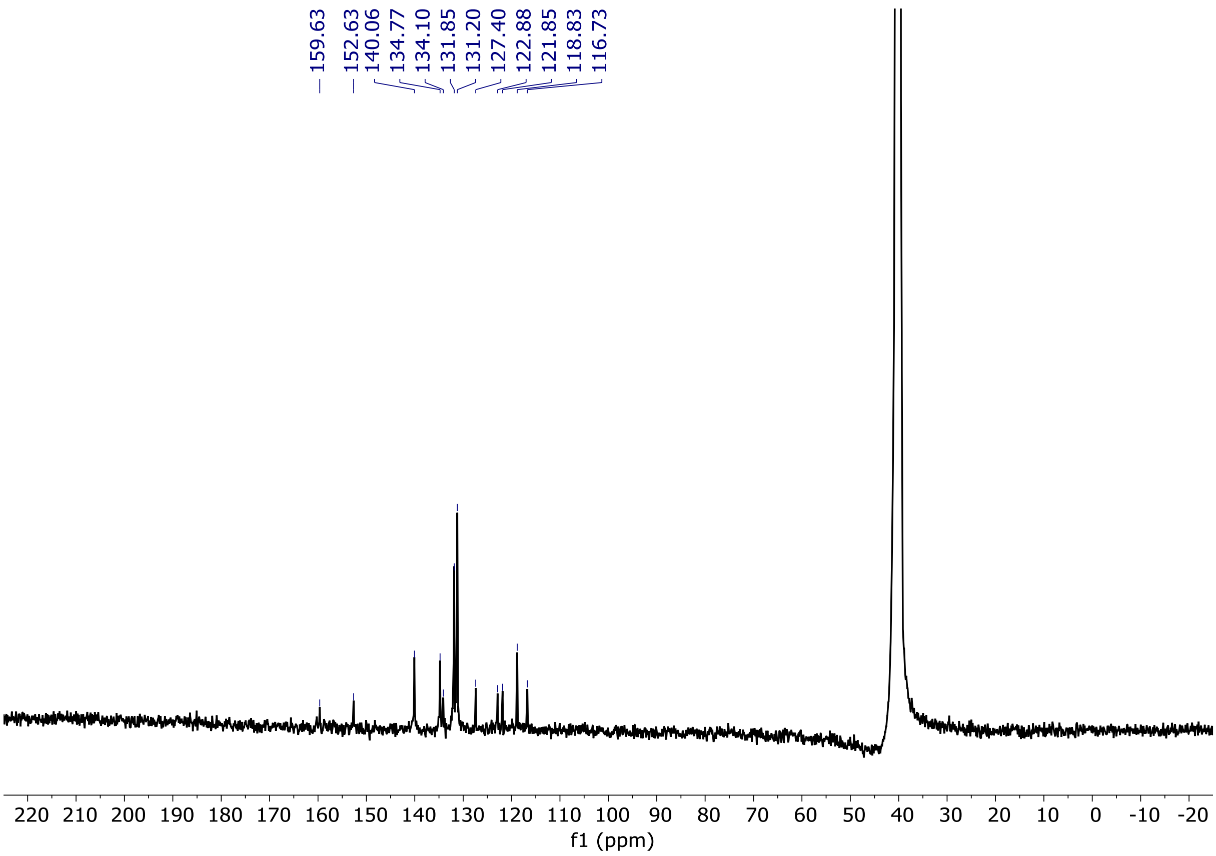


**Figure S4.** $^{13}C$-NMR spectrum of 6-bromo-3-(*p*-bromophenyl)coumarin (**2**).

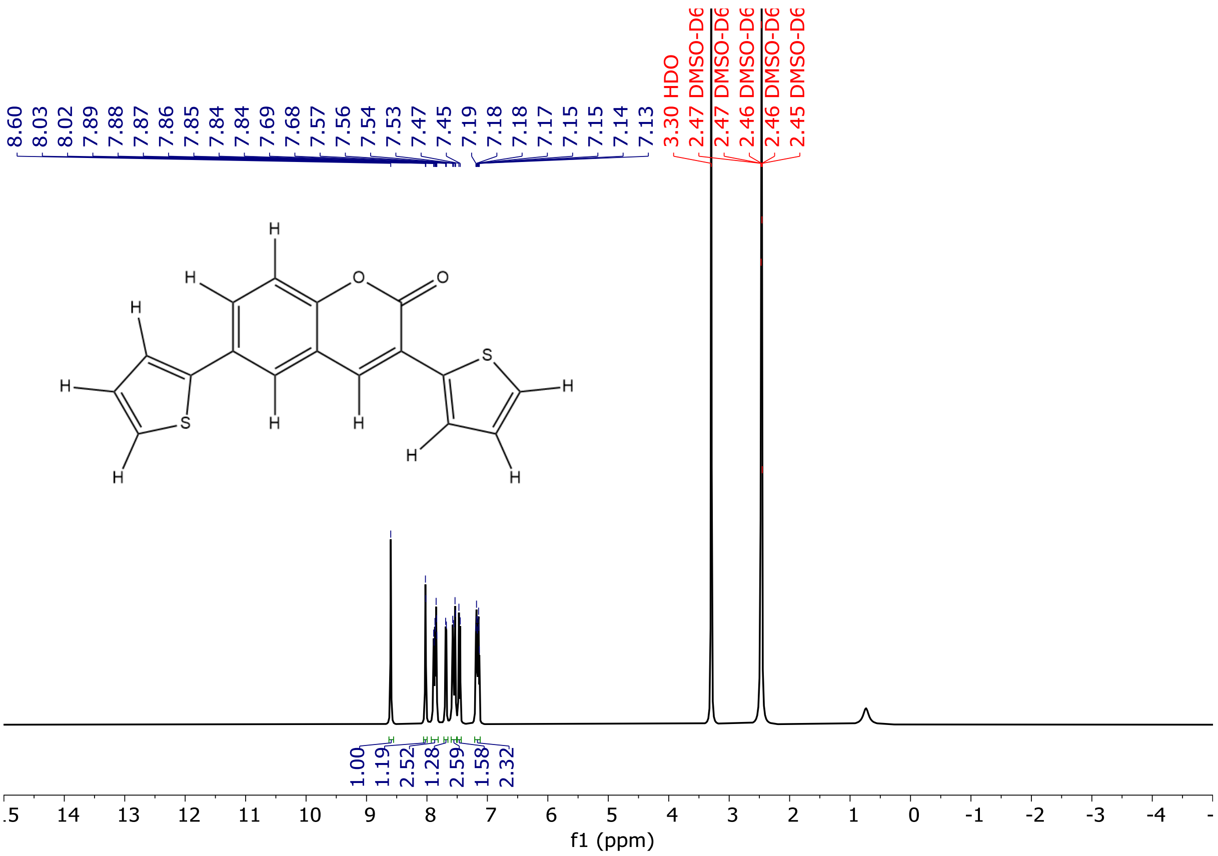


**Figure S5.** $^{1}$H-NMR spectrum of 3,6-dithienylcoumarin (**DTQ**).

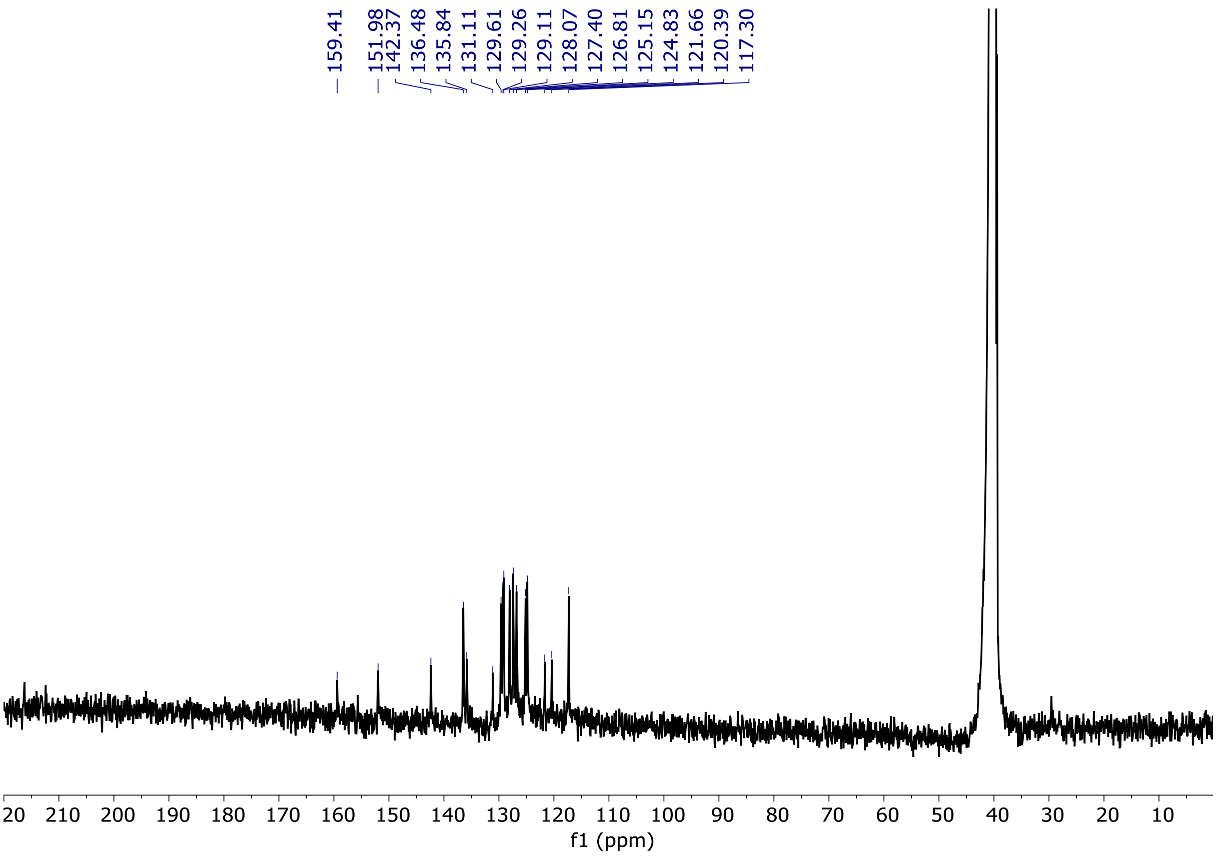


**Figure S6.** $^{13}$C-NMR spectrum of 3,6-dithienylcoumarin (**DTQ**).

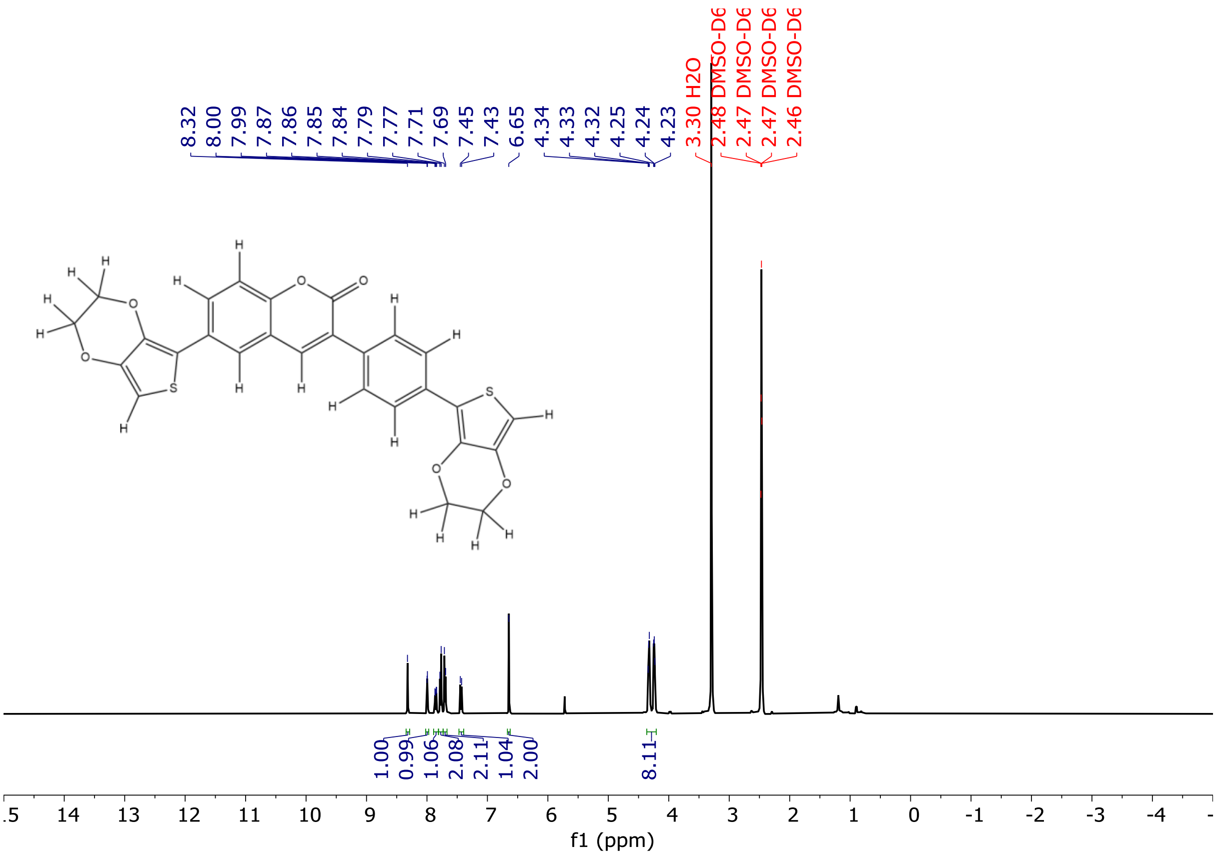

**Figure S7.** $^1$H-NMR spectrum of 6-(2,3-dihydrothieno[3,4,b]dioxinyl)-3-(p-2,3-dihydrothieno[3,4,b]dioxinyl)phenylcoumarin (**EPQ**).

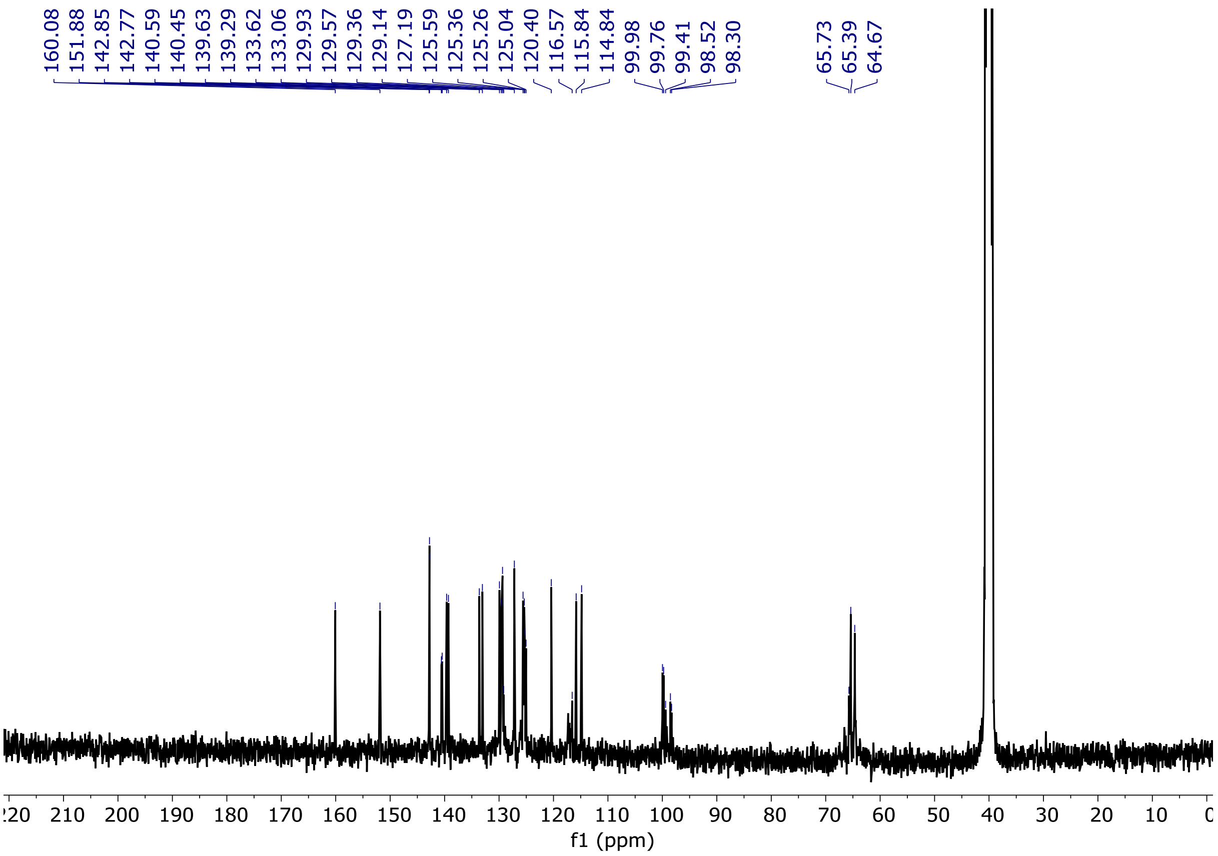


**Figure S8.** $^{13}$C-NMR spectrum of 6-(2,3-dihydrothieno[3,4,b]dioxinyl)-3-(p-2,3-dihydrothieno[3,4,b]dioxinyl)phenylcoumarin (**EPQ**).

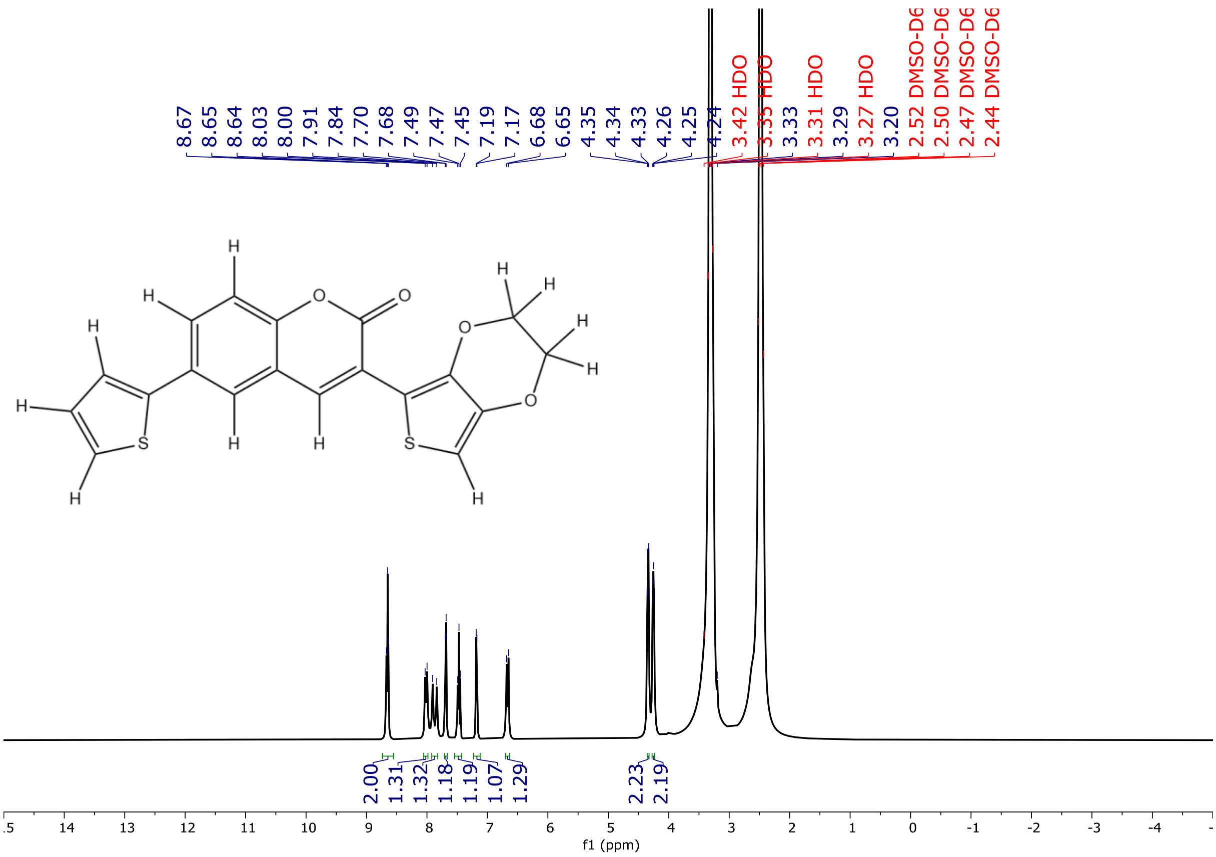


**Figure S9.** $^{1}$H-NMR spectrum of 6-(2,3-dihydrothieno[3,4,b]dioxinyl)-3-thienylcoumarin (**ETQ**).

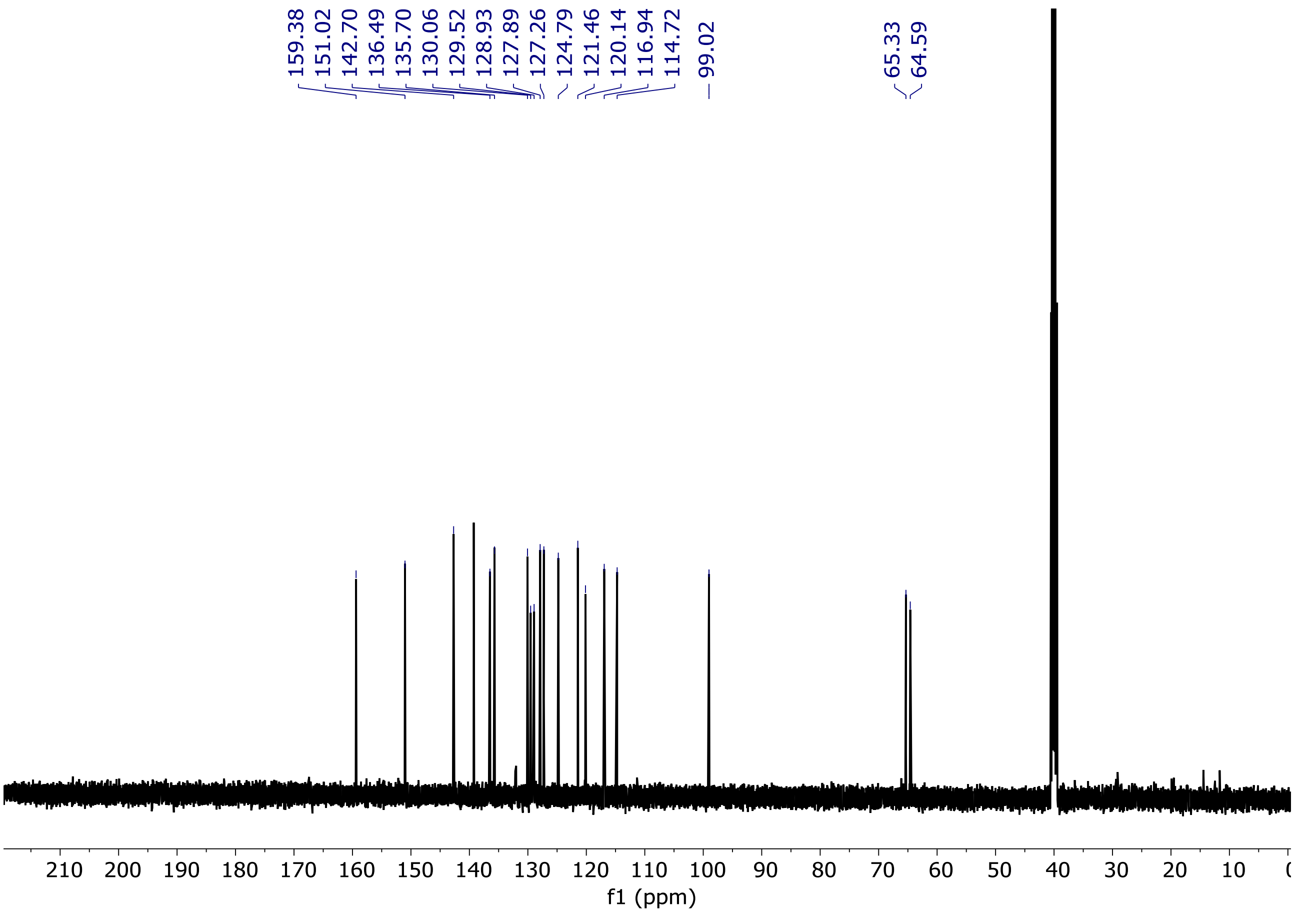


**Figure S10.** $^{13}C$- NMR spectrum of 6-(2,3-dihydrothieno[3,4,b]dioxinyl)-3-thienylcoumarin (**ETQ**).

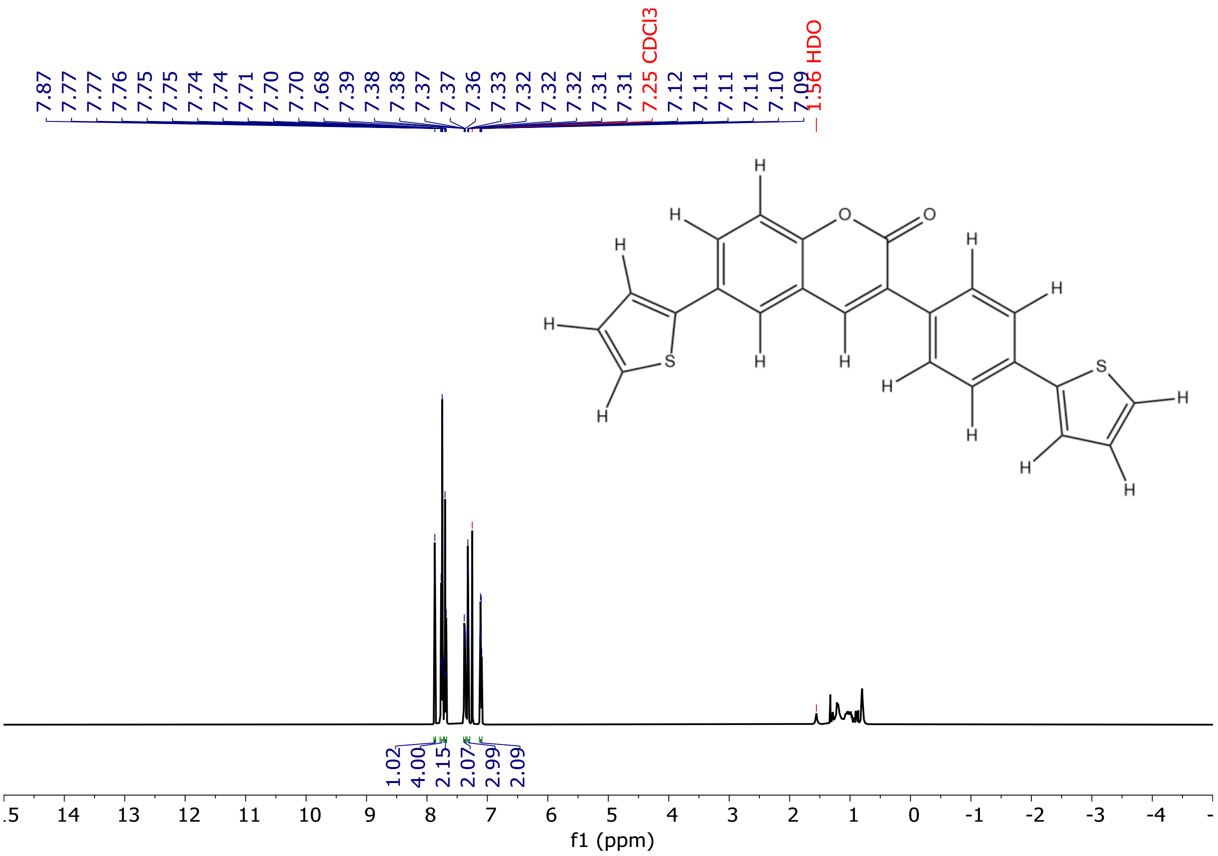

**Figure S11.** $^1$H-NMR spectrum of 6-thienyl-3-(p-thienylphenyl)coumarin (**TPQ**).

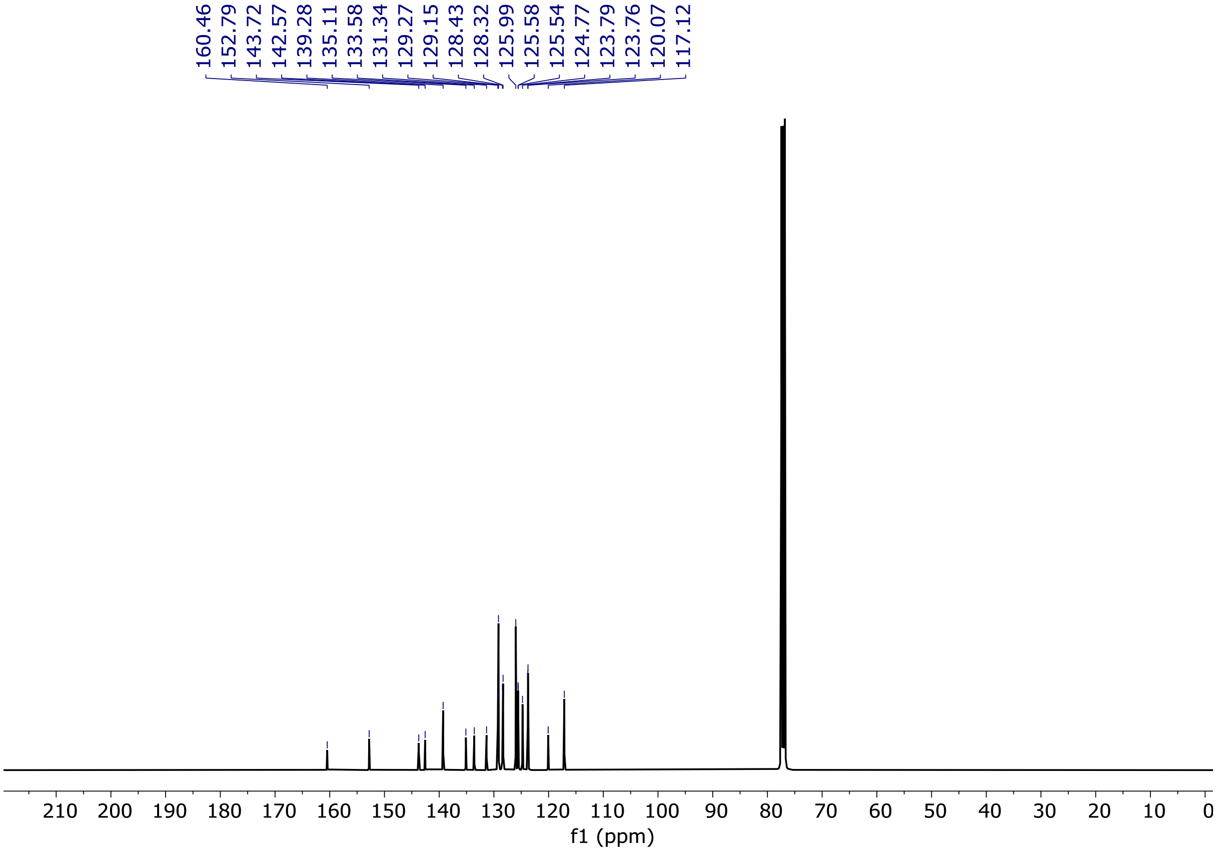


**Figure S12.** $^{13}C$-NMR spectrum of 6-thienyl-3-(p-thienylphenyl)coumarin (**TPQ**).

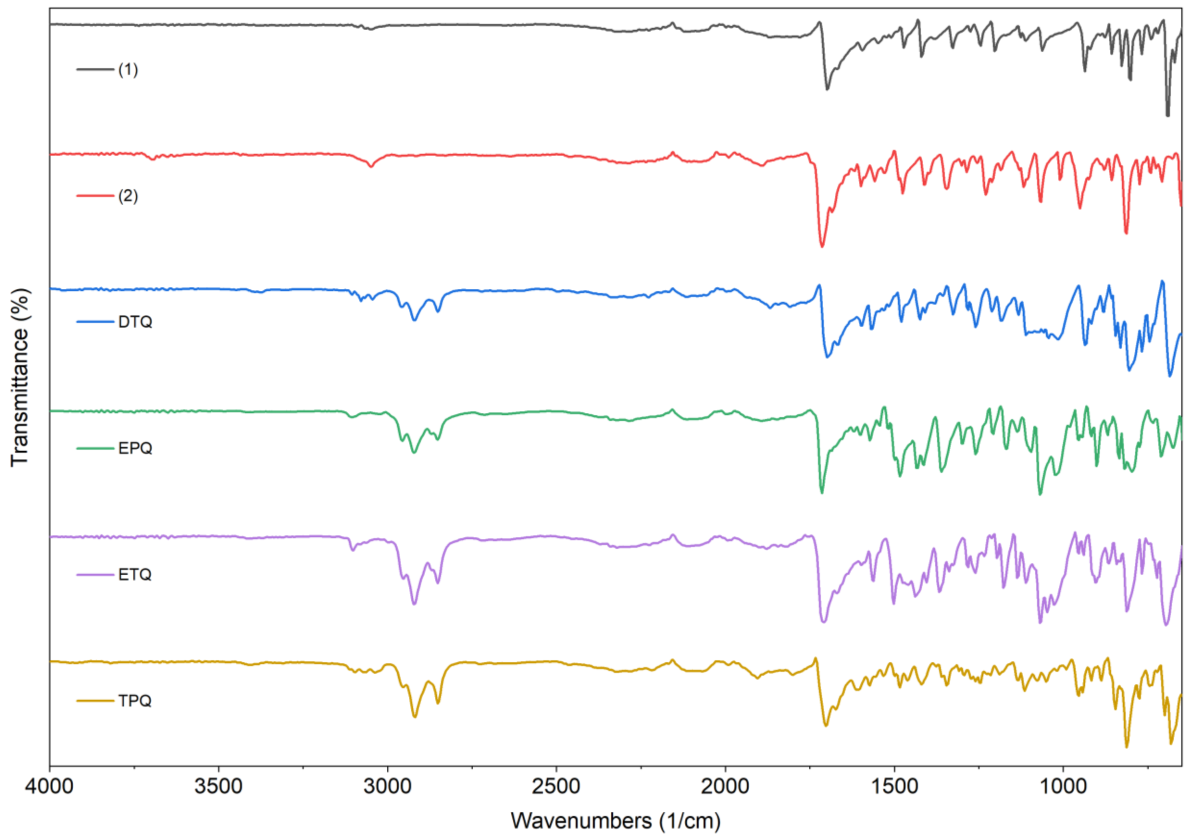


**Figure S13.** The FT-IR spectra.of thiophene-coumarin compounds

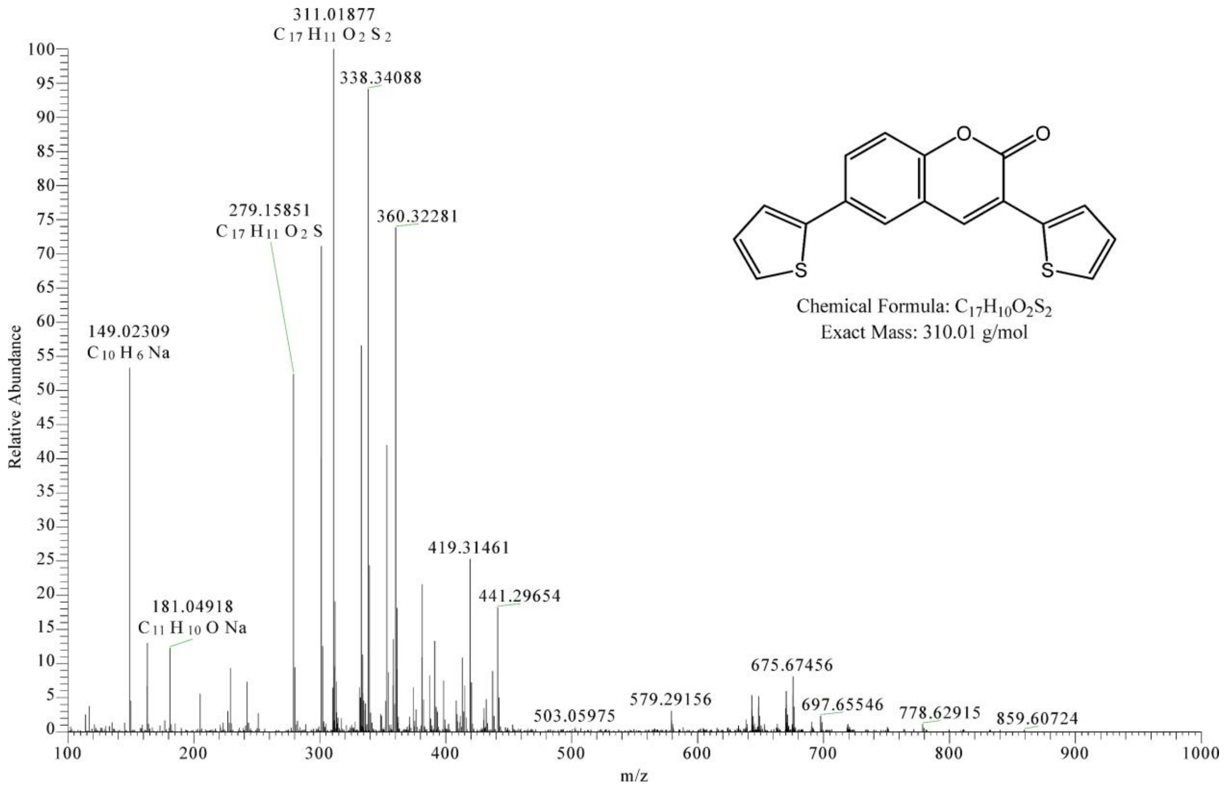


**Figure S14.** The HRMS spectrum of 3,6-dithienylcoumarin (**DTQ**).

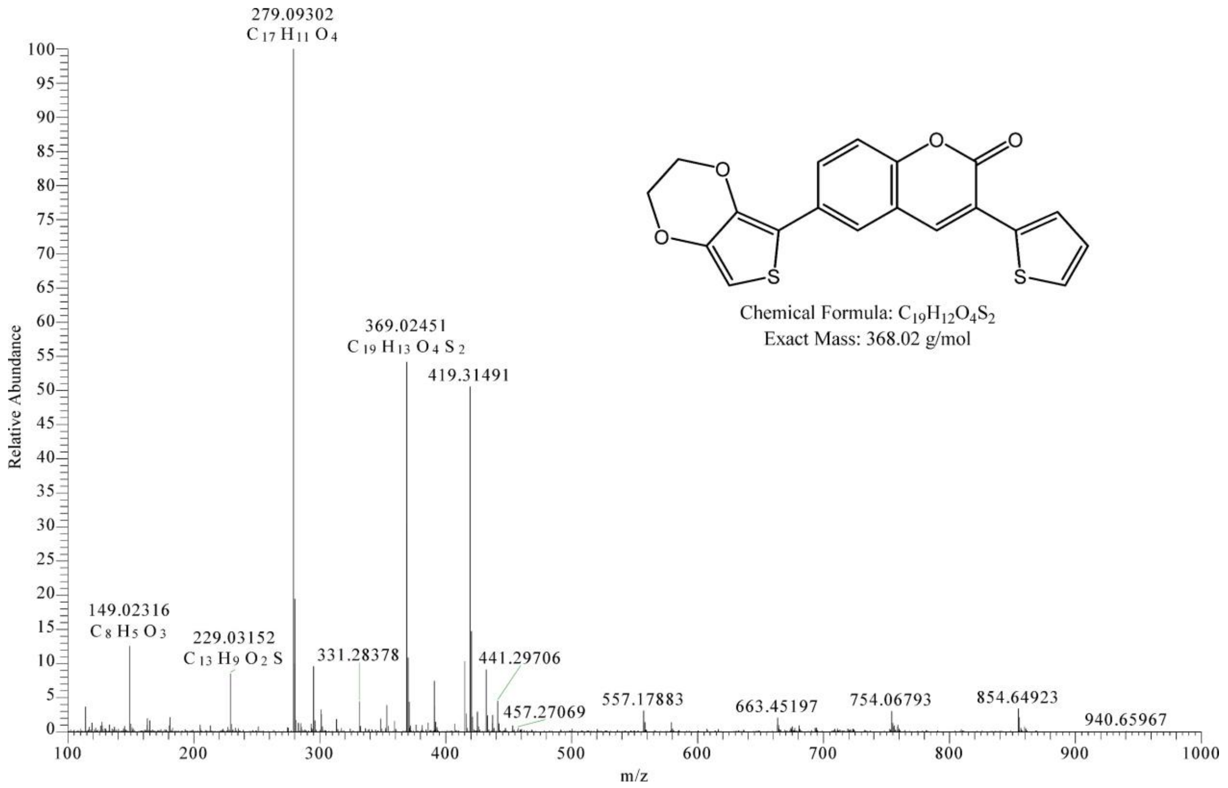


**Figure S15.** The HRMS spectrum of 6-(2,3-dihydrothieno[3,4,b]dioxinyl)-3-thienylcoumarin (ETQ).

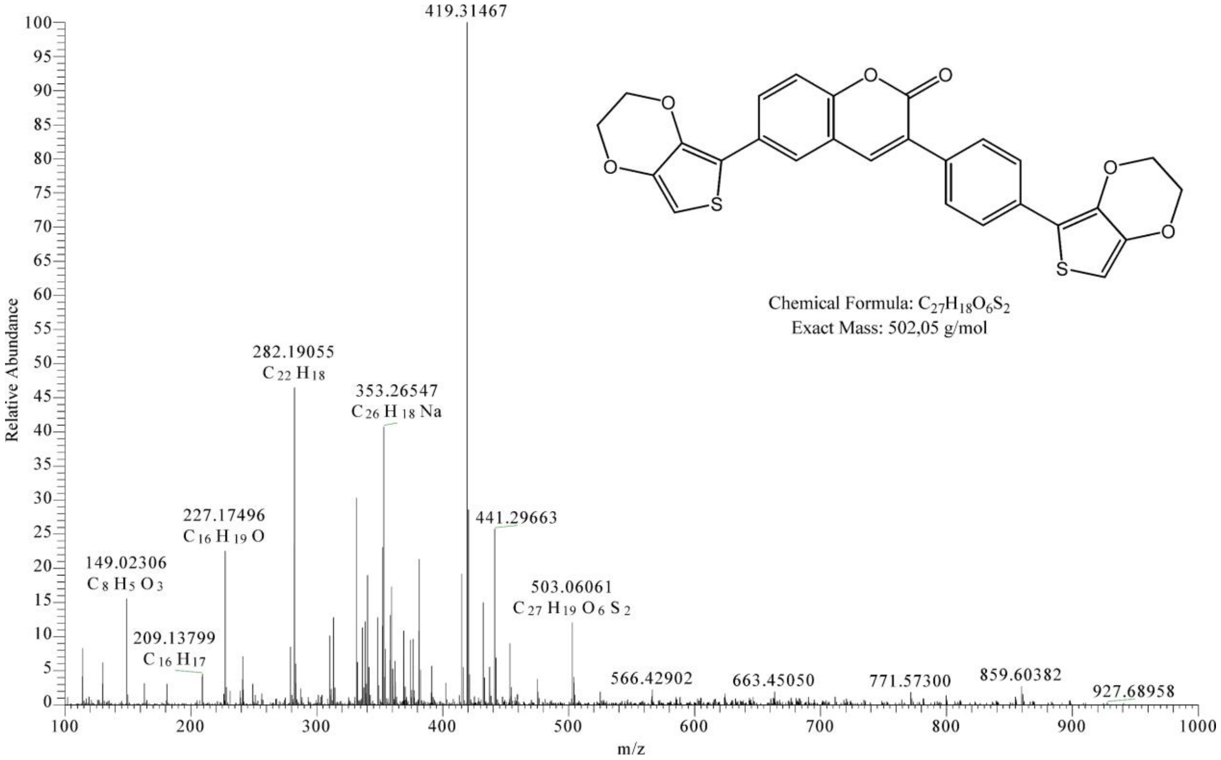


**Figure S16.** The HRMS spectrum of 6-(2,3-dihydrothieno[3,4,b]dioxinyl)-3-(p-2,3-dihydrothieno[3,4,b]dioxinyl)phenylcoumarin (**EPQ**).

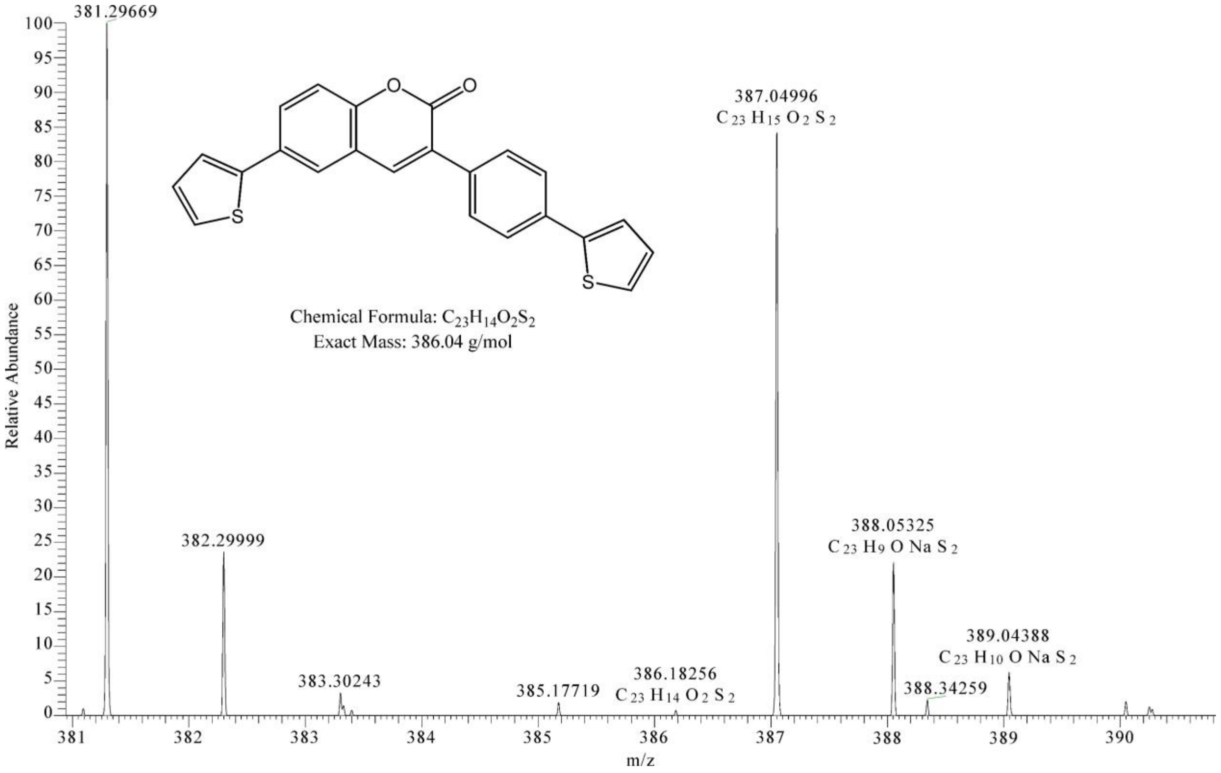


**Figure S17.** The HRMS spectrum of 6-thienyl-3-(p-thienylphenyl)coumarin (**TPQ**).

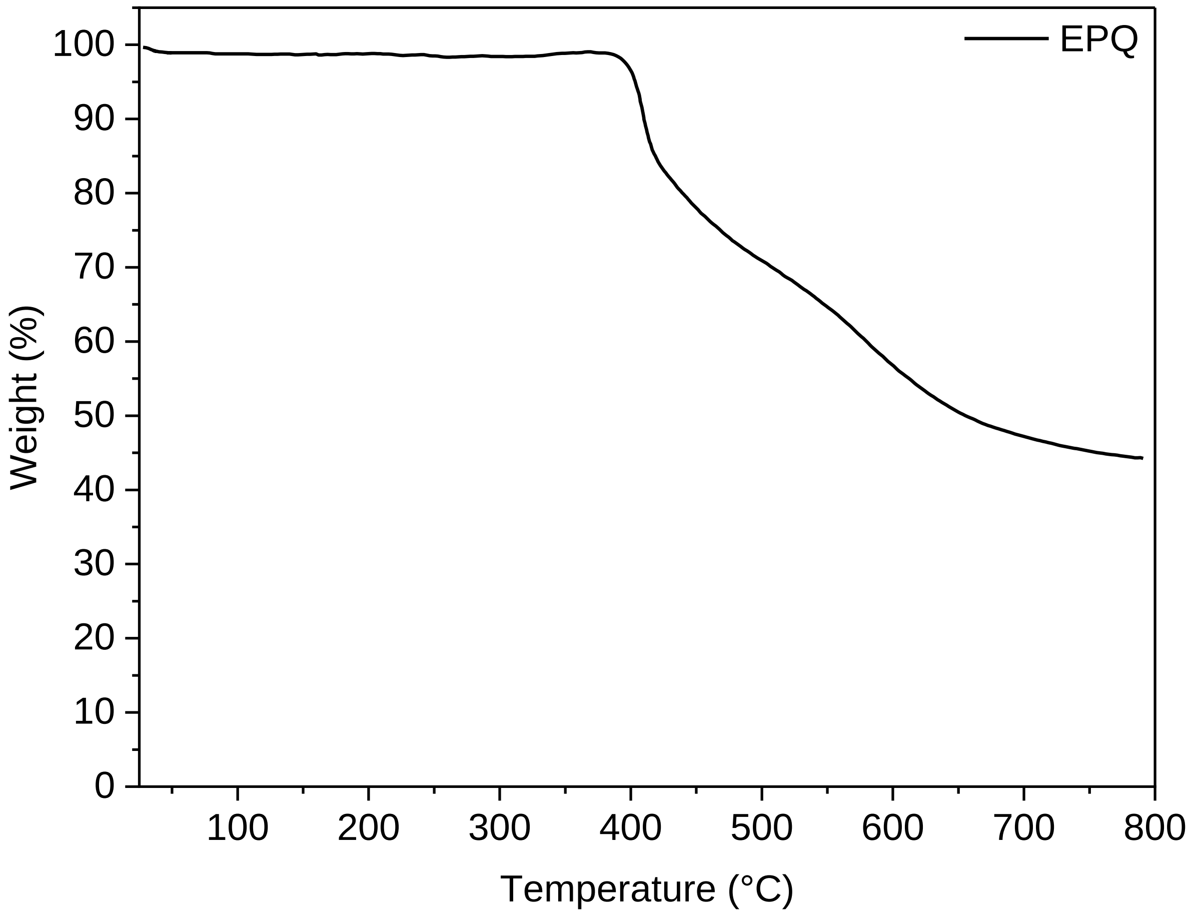


**Figure S18.** TGA spectrum of 6-(2,3-dihydrothieno[3,4,b]dioxinyl)-3-(p-2,3-dihydrothieno[3,4,b]dioxinyl)phenylcoumarin (**EPQ**).

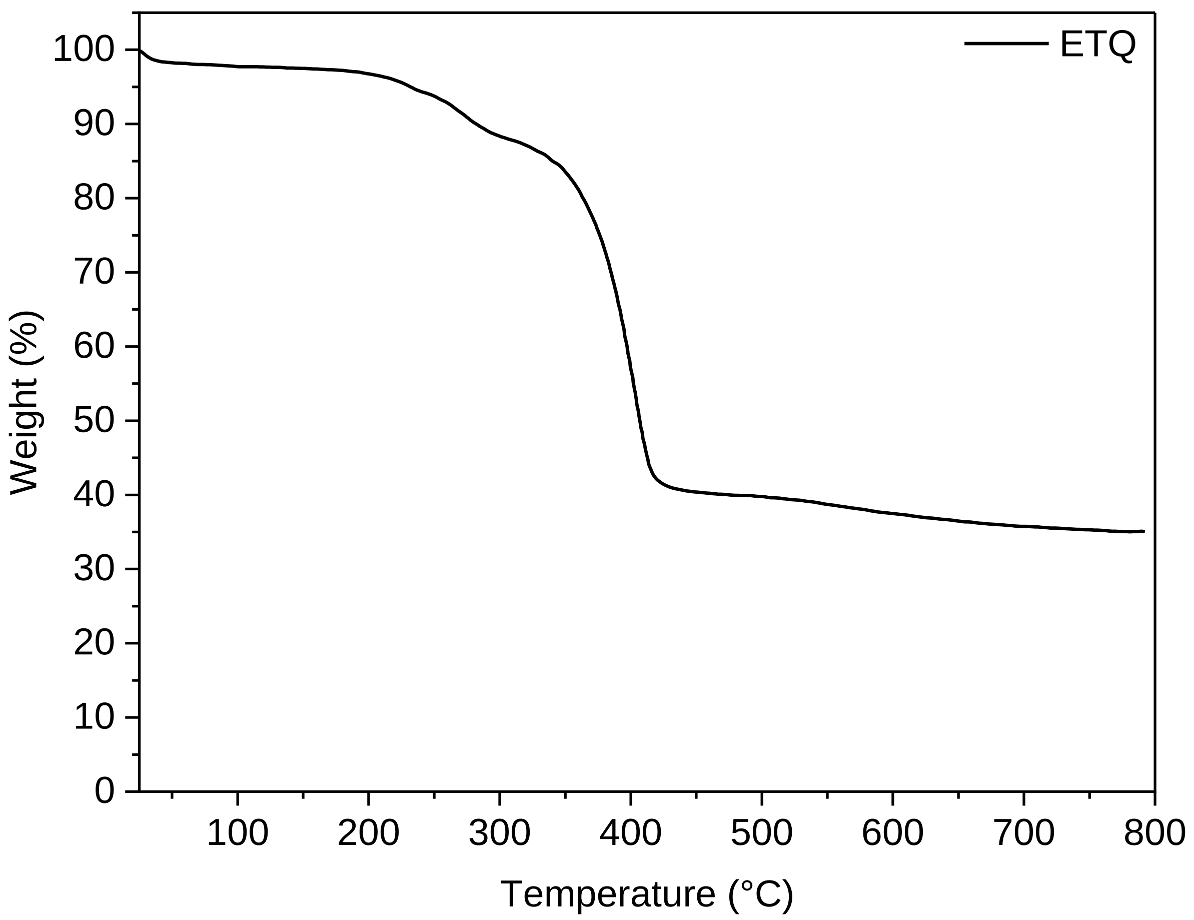


**Figure S19.** TGA spectrum of 6-(2,3-dihydrothieno[3,4,b]dioxinyl)-3-thienylcoumarin (**ETQ**).

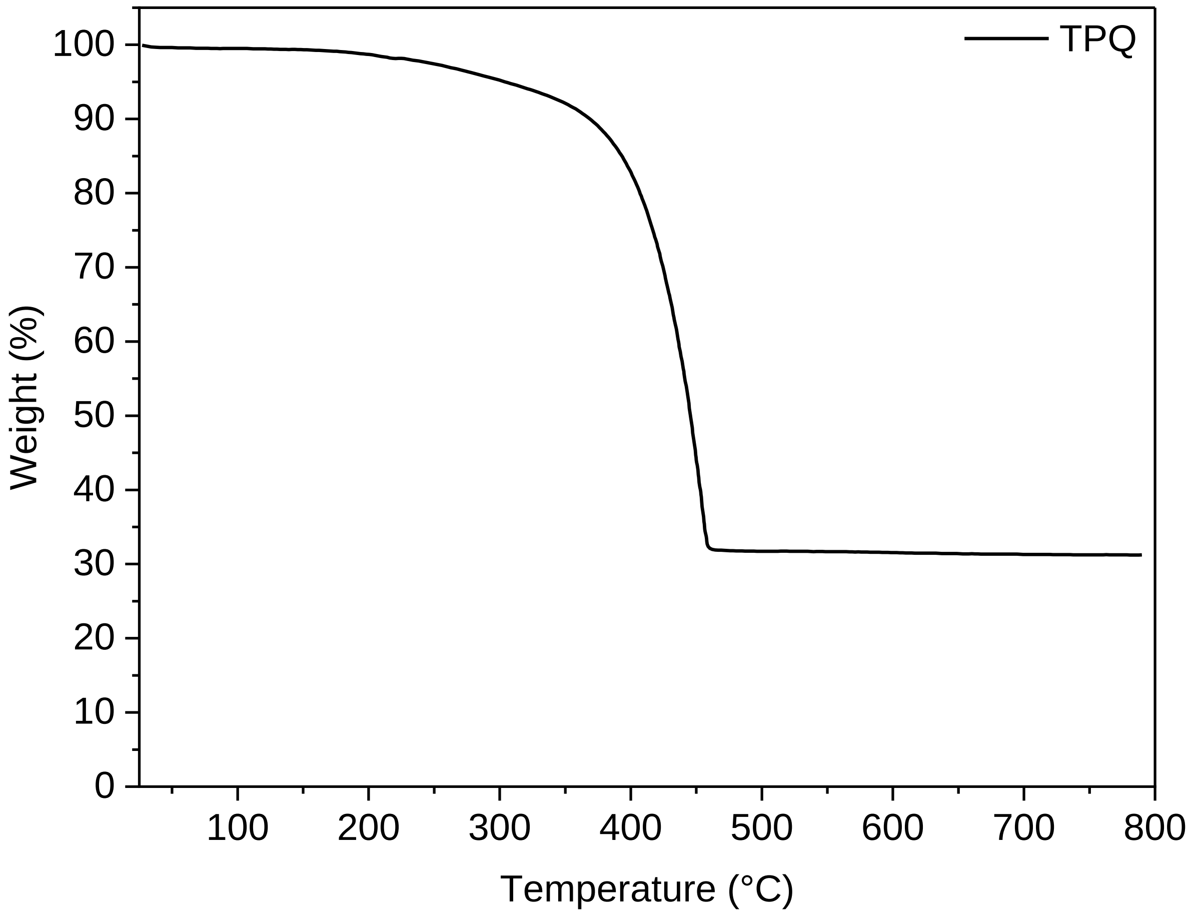


**Figure S20.** TGA spectrum of 6-thienyl-3-(p-thienylphenyl)coumarin (**TPQ**).

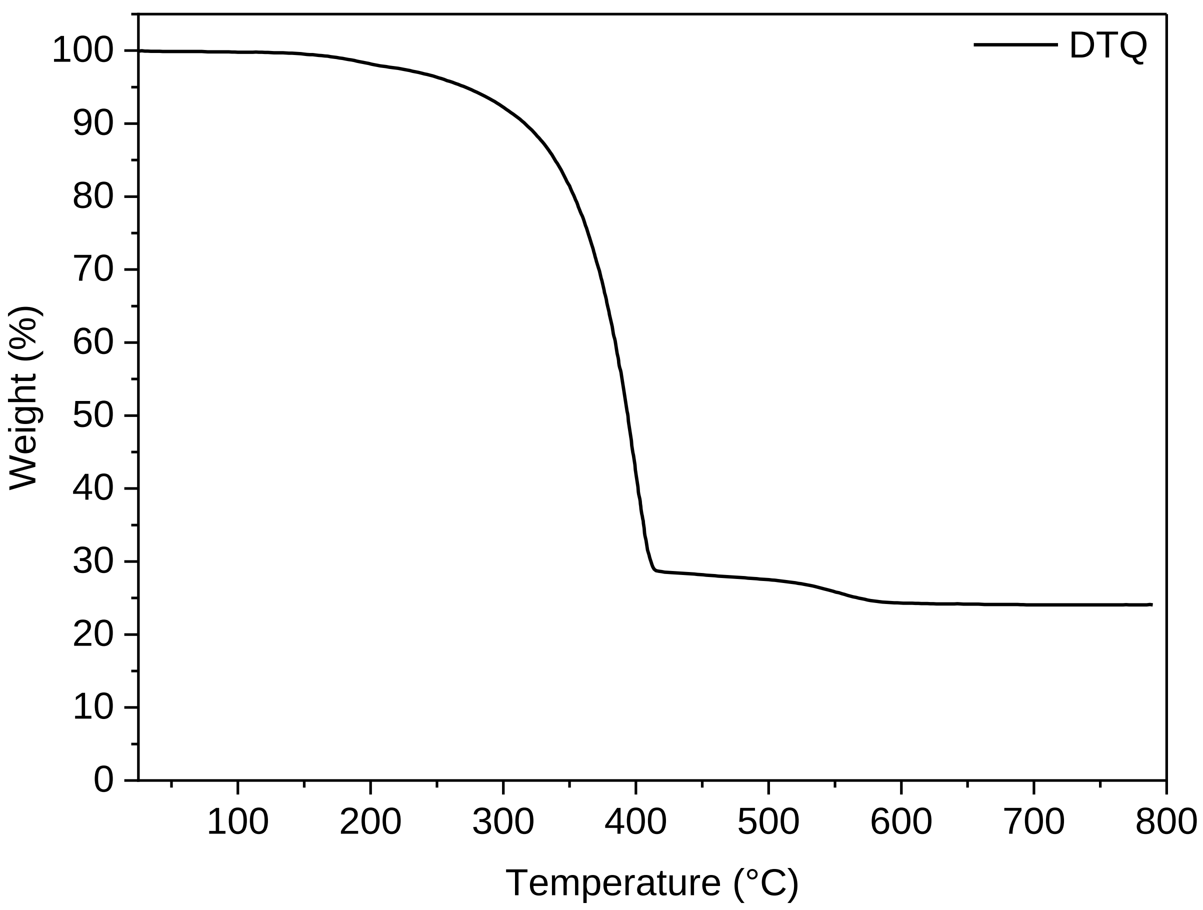


**Figure S21.** TGA spectrum of 3,6-dithienylcoumarin (**DTQ**).

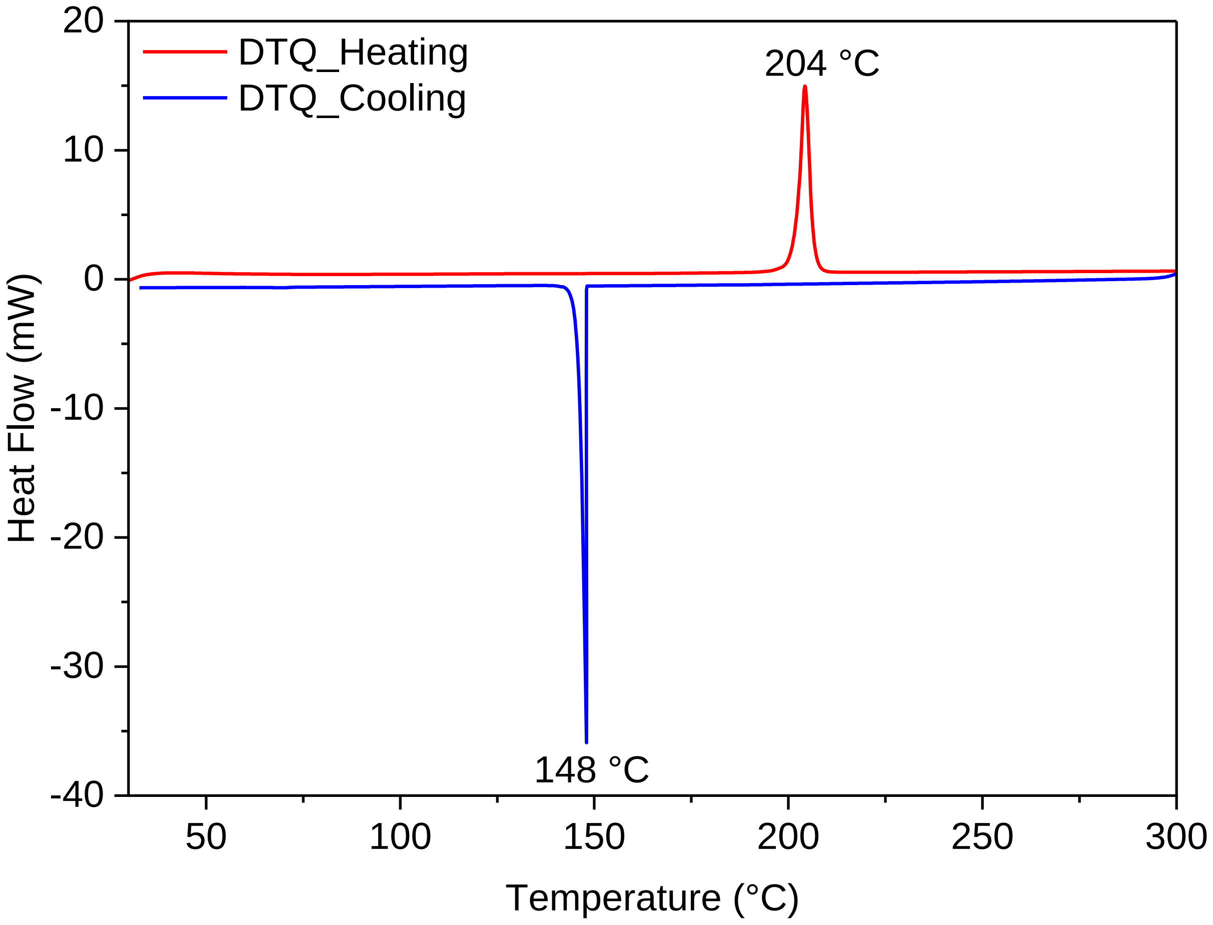


**Figure S22.** DSC spectrum of 3,6-dithienylcoumarin (**DTQ**).

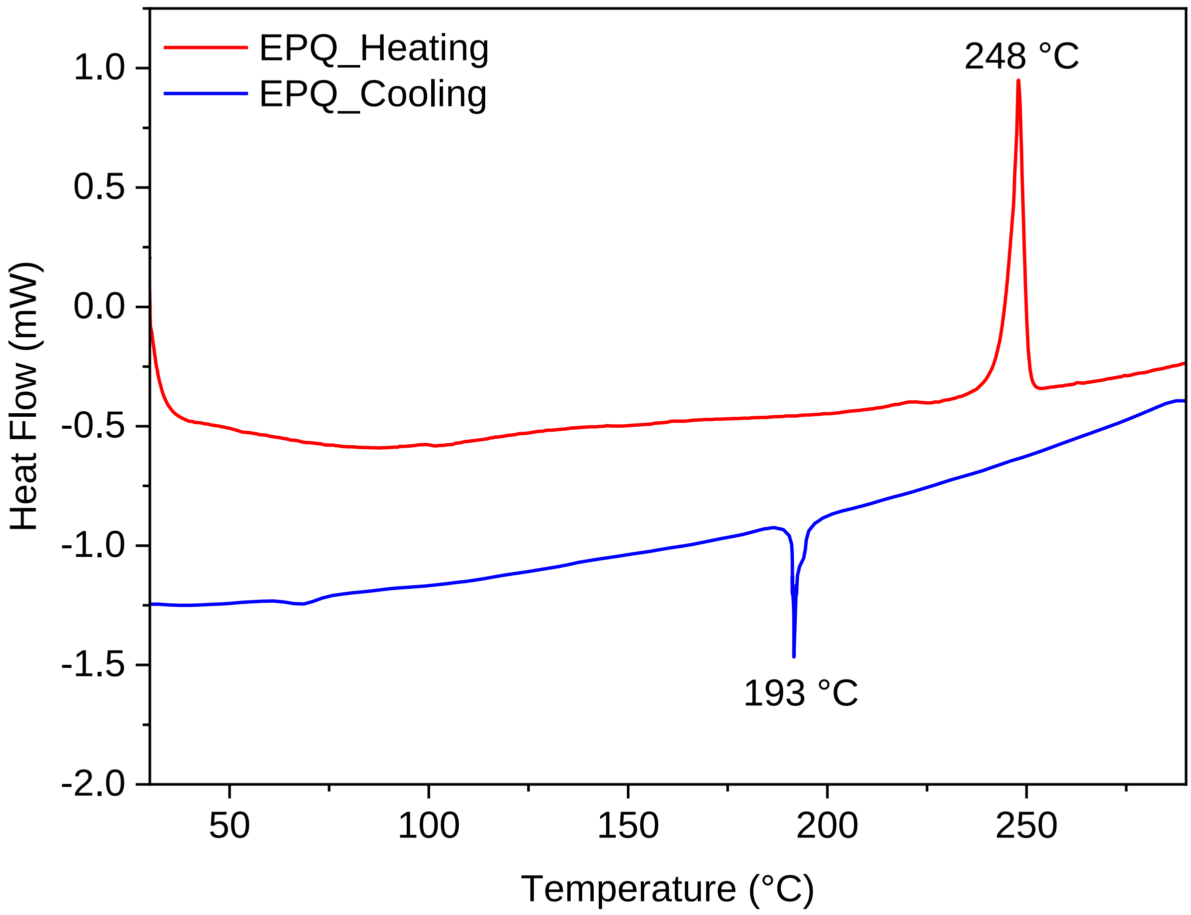


**Figure S23.** DSC spectrum of 6-(2,3-dihydrothieno[3,4,b]dioxinyl)-3-(p-2,3-dihydrothieno[3,4,b]dioxinyl)phenylcoumarin (**EPQ**).

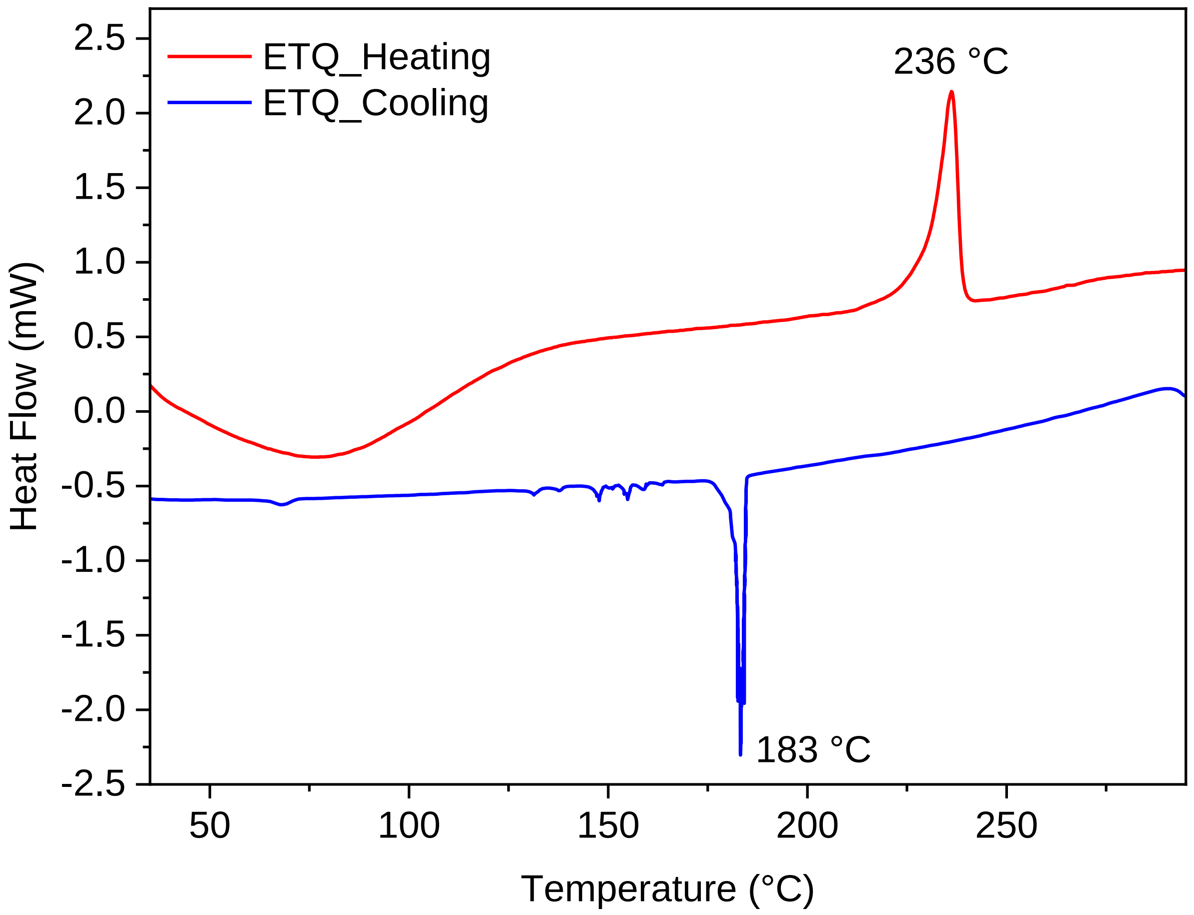


**Figure S24.** DSC spectrum of 6-(2,3-dihydrothieno[3,4,b]dioxinyl)-3-thienylcoumarin (**ETQ**).

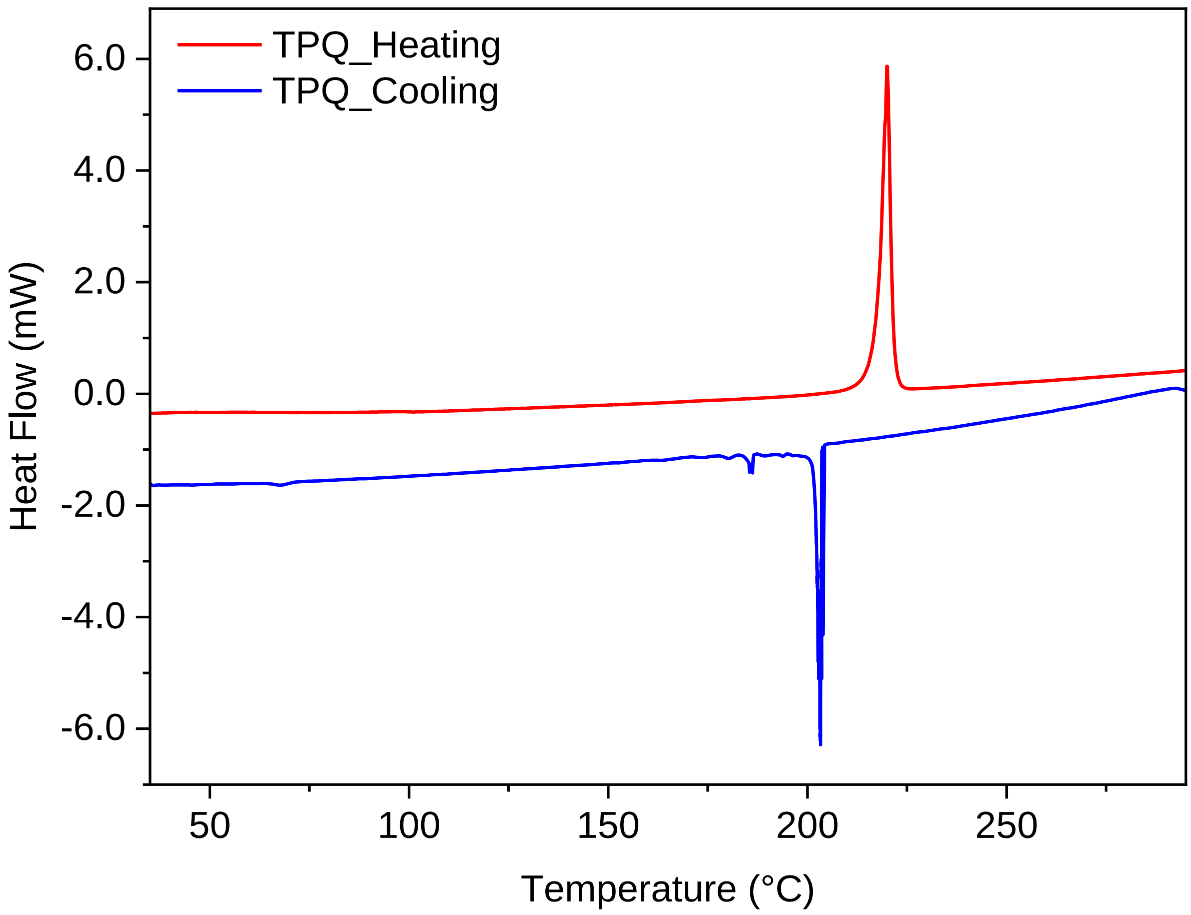


**Figure S25.** DSC spectrum of 6-thienyl-3-(p-thienylphenyl)coumarin (**TPQ**).

**Table S1.** Crystallographic data and refinement parameters for **ETQ, DTQ, TPQ,** and **EPQ**.

| Identification code | ETQ | DTQ | TPQ | EPQ |
|---|---|---|---|---|
| **CCDC** | 2256774 | 2256773 | 2256771 | 2256772 |
| **Empirical formula** | $C_{19}H_{12}O_4S_2$ | $C_{17}H_{10}O_2S_2$ | $C_{23}H_{14}O_2S_2$ | $C_{27}H_{18}O_6S_2$ |
| **Formula weight** | 368.41 | 310.37 | 386.46 | 502.53 |
| **Temperature/K** | 298 | 298 | 298 | 298 |
| **Crystal system** | triclinic | orthorhombic | monoclinic | triclinic |
| **Space group** | P-1 | Pbca | $P2_1$ | P-1 |
| **a/Å** | 4.729(2) | 13.264(10) | 7.5038(10) | 8.0067(13) |
| **b/Å** | 11.303(5) | 9.169(7) | 13.1909(18) | 13.887(2) |
| **c/Å** | 15.710(7) | 22.538(17) | 18.094(3) | 21.062(3) |
| **α/°** | 71.082(10) | 90 | 90 | 99.172(3) |
| **β/°** | 85.537(10) | 90 | 92.262(3) | 97.379(3) |
| **γ/°** | 82.843(11) | 90 | 90 | 105.221(3) |
| **Volume/Å$^3$** | 787.5(6) | 2741(4) | 1789.6(4) | 2194.8(6) |
| **Z** | 2 | 8 | 4 | 4 |
| **$\rho_{calc}$g/cm$^3$** | 1.554 | 1.504 | 1.434 | 1.521 |
| **μ/mm$^{-1}$** | 0.361 | 0.388 | 0.313 | 0.288 |
| **F(000)** | 380.0 | 1280.0 | 800.0 | 1040.0 |
| **Crystal size/mm$^3$** | 0.244 × 0.075 × 0.044 | 0.578 × 0.129 × 0.084 | 0.293 × 0.218 × 0.081 | 0.181 × 0.131 × 0.082 |
| **2Θ range for data collection/°** | 7.274 to 50.05 | 3.614 to 50.06 | 3.822 to 50.162 | 3.104 to 49.99 |
| **Index ranges** | -5 ≤ h ≤ 5, -13 ≤ k ≤ 13, -18 ≤ l ≤ 18 | -15 ≤ h ≤ 15, -10 ≤ k ≤ 10, -26 ≤ l ≤ 26 | -8 ≤ h ≤ 8, -15 ≤ k ≤ 15, -21 ≤ l ≤ 21 | -9 ≤ h ≤ 9, -16 ≤ k ≤ 16, -25 ≤ l ≤ 25 |
| **Reflections collected** | 8381 | 16615 | 22722 | 27823 |
| **Independent reflections** | 2781 [$R_{int}$ = 0.2175, $R_{sigma}$ = 0.3472] | 2416 [$R_{int}$ = 0.0857, $R_{sigma}$ = 0.0503] | 6316 [$R_{int}$ = 0.0673, $R_{sigma}$ = 0.0790] | 7734 [$R_{int}$ = 0.0777, $R_{sigma}$ = 0.0804] |
| **Data/restraints/parameters** | 2781/431/291 | 2416/0/190 | 6316/233/599 | 7734/680/707 |
| **Goodness-of-fit on F$^2$** | 0.932 | 1.017 | 1.016 | 1.009 |
| **Final R indexes [I>=2σ (I)]** | $R_1$ = 0.0974, $wR_2$ = 0.2004 | $R_1$ = 0.0483, $wR_2$ = 0.1188 | $R_1$ = 0.0627, $wR_2$ = 0.1521 | $R_1$ = 0.0613, $wR_2$ = 0.1426 |
| **Final R indexes [all data]** | $R_1$ = 0.3087, $wR_2$ = 0.2880 | $R_1$ = 0.0811, $wR_2$ = 0.1369 | $R_1$ = 0.1222, $wR_2$ = 0.1817 | $R_1$ = 0.1348, $wR_2$ = 0.1636 |
| **Largest diff. peak/hole / e Å$^{-3}$** | 0.46/-0.30 | 0.24/-0.39 | 0.51/-0.24 | 0.33/-0.25 |
